# Disrupted propionate metabolism evokes transcriptional changes in the heart by increasing histone acetylation and propionylation

**DOI:** 10.1038/s44161-023-00365-0

**Published:** 2023-11-23

**Authors:** Kyung Chan Park, Nicholas T. Crump, Niamh Louwman, Steve Krywawych, Yuen Jian Cheong, Iolanda Vendrell, Eleanor K. Gill, Mala Gunadasa-Rohling, Kerrie L. Ford, David Hauton, Marjorie Fournier, Elisabete Pires, Lydia Watson, Gerald Roseman, James Holder, Andreas Koschinski, Ricardo Carnicer, M. Kate Curtis, Manuela Zaccolo, Alzbeta Hulikova, Roman Fischer, Holger B. Kramer, James S. O. McCullagh, Sophie Trefely, Thomas A. Milne, Pawel Swietach

**Affiliations:** 1 Department of Physiology, Anatomy & Genetics, University of Oxford, Oxford, UK; 2 MRC Molecular Haematology Unit, Radcliffe Department of Medicine, MRC Weatherall Institute of Molecular Medicine, University of Oxford, Oxford, UK; 3 Department of Chemical Pathology, Great Ormond Street Hospital NHS Foundation Trust, London, UK; 4 Epigenetics & Signalling Programmes, Babraham Institute, Cambridge, UK; 5 Nuffield Department of Medicine, Target Discovery Institute, Oxford, UK; 6 Nuffield Department of Medicine, Chinese Academy for Medical Sciences Oxford Institute, University of Oxford, Oxford, UK; 7 Department of Chemistry, University of Oxford, Oxford, UK; 8 Department of Biochemistry, University of Oxford, Oxford, UK; 9 Division of Cardiovascular Medicine, Radcliffe Department of Medicine, University of Oxford, Oxford, UK; 10 MRC Laboratory of Molecular Biology, Cambridge Biomedical Campus, Cambridge, UK

## Abstract

Propiogenic substrates and gut bacteria produce propionate, a post-translational protein modifier. In this study, we used a mouse model of propionic acidaemia (PA) to study how disturbances to propionate metabolism result in histone modifications and changes to gene expression that affect cardiac function. Plasma propionate surrogates were raised in PA mice, but female hearts manifested more profound changes in acyl-CoAs, histone propionylation and acetylation, and transcription. These resulted in moderate diastolic dysfunction with raised diastolic Ca^2+^, expanded end-systolic ventricular volume and reduced stroke volume. Propionate was traced to histone H3 propionylation and caused increased acetylation genome-wide, including at promoters of *Pde9a* and *Mme*, genes related to contractile dysfunction through downscaled cGMP signaling. The less severe phenotype in male hearts correlated with β-alanine buildup. Raising β-alanine in cultured myocytes treated with propionate reduced propionyl-CoA levels, indicating a mechanistic relationship. Thus, we linked perturbed propionate metabolism to epigenetic changes that impact cardiac function.

Post-translational modifications (PTMs) of histone lysine residues can change chromatin structure and gene transcription^1^. A well-studied histone modification is acetylation^2^, which plays a prominent role in cardiac development^3^, responses to environmental triggers^4^ and cardiac disease^5,6^. Propionate, the three-carbon homologue of acetate, can also modify histones, but its metabolic supply lines are more restricted, and less is known about its significance in the heart. Sources of propionate include the catabolism of odd-numbered fatty acids, methionine and branched-chain amino acids (BCAAs)^7^ as well as the activity of gut bacteria^8^, but whether this is sufficient to evoke epigenetic changes in the heart remains elusive. Propionyl-CoA has been shown to propionylate histones in cultured cells^9,10^ and in the liver^11^ and brain^12^ in vivo, although its cardiac actions remain unclear. Propionyl-CoA may also increase histone acetylation by activating the acetyltransferase p300 (ref. 13) or through the inhibitory effect of propionate on histone deacetylases (HDACs), akin to that of butyrate^8,14,15^.

The link between propionate and cardiac dysfunction can be interrogated by studying propionic acidaemia (PA), an inborn error of metabolism. In PA, loss-of-function mutations in *PCCA* or *PCCB* result in reduced or absent propionyl-CoA carboxylase (PCC) activity^7^, which hinders propionyl-CoA catabolism. Increased bioavailability of propionyl-CoA/propionate may potentially act as a substrate for^10,16^ or inducer of^14,17,18^ histone acylation^13^. Patients with PA commonly develop cardiac dysfunction, characterised by dilated cardiomyopathy and long-QT arrythmias^19^, which have been discussed in terms of toxicity and mitochondrial dysregulation^20^, but a role for epigenetic changes cannot be excluded. Establishing a link between propionyl-CoA and gene expression would add a new and important axis to our understanding of cardiometabolic disease, ranging from other inborn errors of metabolism^21^ (for example, methylmalonic acidaemia)^19^ to diabetes^22^.

We studied mice with genetically altered PCC activity (FVB *Pcca*^−/−^ A138T)^23^, wherein the murine *Pcca* gene was knocked out and a hypomorphic human mutant (*PCCA* A138T) was inserted. This hypomorphic model maintains an elevated propionyl-CoA load and has been used to study a mild to moderate form of PA, because, unlike complete knockouts^24^, it supports growth into adult life^23^, which is essential for understanding phenotypes at the organism level. Previous studies described this mouse as having dysregulated Ca^2+^ handling and diastolic impairment at 8 months of age, attributing these changes to cumulative oxidative damage and microRNA dysregulation^25^. In the present study, we used young adult mice to investigate epigenetic responses, because, by this age, PA markers^26^ stabilise at an elevated level, and epigenetic changes are expected, but without the confounding effects of oxidative damage that emerge in later life^27^. We found that a global genetic disruption to propionate metabolism raises cardiac propionate and propionyl-CoA to levels that cause histone propionylation and a net increase in acetylation in vivo. These PTMs are sufficient to trigger a transcriptional response, including the upregulation of genes previously implicated in cardiac dysfunction. We report here a sex dependence in terms of metabolic disturbance that produced a proportionally greater transcriptional and phenotypic response in females. The smaller propionyl-CoA excess in male PA hearts related to higher levels of β-alanine, a rate-limiting precursor of carnosine that serves as a major antioxidant in the heart^28^. We speculate a role for these actions in hearts under metabolic stresses that influence propionate metabolism.

## Results

### Surrogates of propionate metabolism are raised in PA mice

**Fig. 1:**
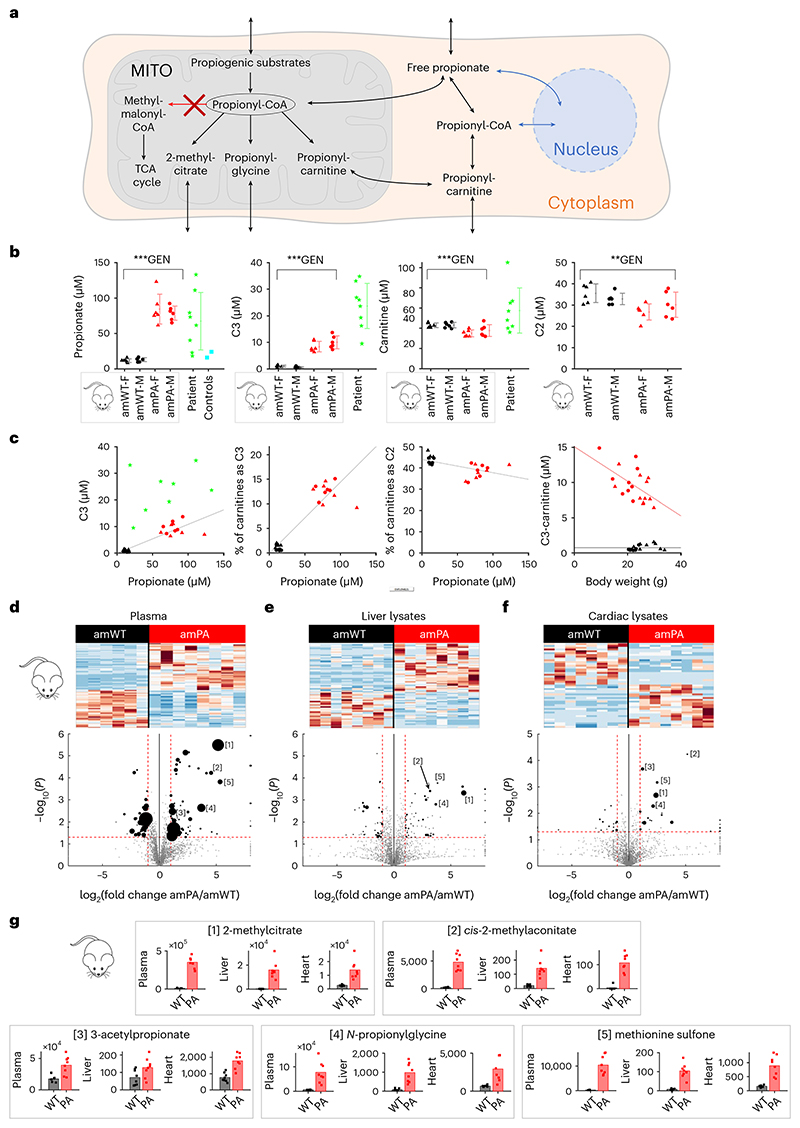
Metabolic consequences of disrupted PCC activity. **a**, Selected metabolic pathways processing propionate. Propiogenic substrates (for example, BCAAs, odd-numbered fatty acids, methionine and cholesterol) yield propionyl-CoA in the mitochondrial (MITO) matrix. Under restricted recruitment into the Krebs cycle by genetically ablated PCC (red cross), propionyl-CoA is converted to 2-methylcitrate, propionyl-glycine, propionyl-carnitine or other derivatives. Some derivatives interconvert to free propionate that readily crosses membranes in its protonated form. **b**, GC–MS/MS of plasma samples from 8-week female and male mice of WT or PA genotype: free propionate, propionylcarnitine (C3), free carnitine and acetylcarnitine (C2) (two-way ANOVA, significant effect of genotype at **/*** *P* < 0.01/*P* < 0.001; *n* = 6 biologically independent samples). Also shown are data from eight patients with a managed form of PA and two control, non-PA patient samples for reference. Mean ± s.e.m. **c**, Correlations between plasma parameters. Also shown is the relationship (*P* < 0.05, Pearson’s test) between mouse body weight and propionyl-carnitine, with additional mouse samples included that had substantial weight loss. IC–MS of sex-balanced plasma samples (*n* = 6 amWT and *n* = 8 amPA biologically independent samples) (**d**), liver lysates (*n* = 8 amWT and *n* = 8 amPA biologically independent samples) (**e**) and cardiac lysates (*n* = 8 amWT and *n* = 8 amPA biologically independent samples) (**f**). Heat maps show differentially abundant metabolites. Symbol size is proportional to the square root of mean signal intensity. [1] 2-methylcitrate, [2] *cis*-2-methylaconitate, [3] 3-acetylpropionate, [4] *N*-propionylglycine, [5] methionine sulfone. Analysis by one-factor ANOVA (MetaboAnalyst) corrected for multiple comparisons. **g**, IC–MS signal for selected substances detected in plasma, liver and cardiac lysates. *y* axis: intensity. Mean ± s.e.m.

Propionyl-CoA is generated in mitochondria and can be processed to derivatives, including propionyl-carnitine—a commonly used surrogate^26^ (Fig. 1a). These metabolites can interconvert with free propionate anions, which cross membranes as the acid. We used plasma propionate as a proxy of systemic propionate excess to benchmark the PA mouse against human patients. Propionate in decompensated patients with PA can reach low millimolar levels^29–31^, but patients with managed disease have considerably lower levels: 67 (±41) μM propionate and 23 (±8.5) μM propionyl-carnitine (Fig. 1b). Mouse plasma was prepared from blood freshly collected after the induction of terminal anesthesia in 8-week PA mice (‘amPA’). Measurements indicated 81 (±16) μM propionate, which is within the range of patients with PA and substantially elevated compared to 12 (±2.6) μM in wild-type littermates (‘amWT’). Free propionate correlated positively with propionyl-carnitine and negatively with acetyl-carnitine (Fig. 1c). There was no apparent sex dependence in plasma metabolites, arguing for an equivalent global propionate load in male and female PA mice.

### Cardiac tissue generates propionate derivatives

We performed biochemical analyses on plasma and snap-frozen hearts and livers harvested from amPA and amWT mice. Metabolomic analyses by ion-exchange chromatography mass spectrometry (IC–MS) confirmed the metabolic signature of PA in amPA mice: elevated 2-methylcitrate and *N*-propionylglycine^32^ (Fig. 1d,e), including in the heart (Fig. 1f). For example, 2-methylcitrate and *cis*-2-methylaconitate accumulation indicate that propionyl-CoA becomes conjugated to Krebs cycle intermediates in place of acetyl-CoA; raised 3-acetylpropionate and *N*-propionylglycine levels are evidence for chemical conjugation of excess propionate; and the buildup of the oxidized methionine denotes a backlog of this propiogenic amino acid (Fig. 1g). These observations indicate that the PA mouse heart processes considerable propionate fluxes, which may trigger biological responses within the myocyte.

### Propionate metabolism shows sex-dependent differences

**Fig. 2:**
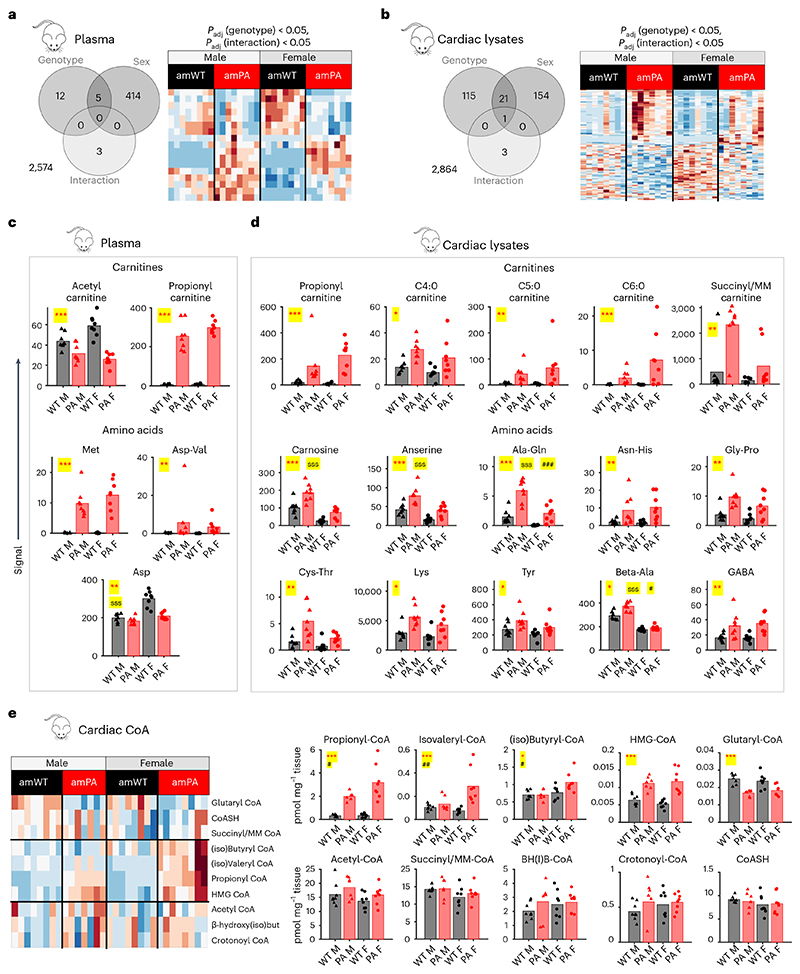
Metabolic consequences of disrupted propionyl-CoA metabolism in the heart. Analysis of ACQ-derivatised amino acids and of underivatised carnitines in plasma (*n* = 8 amWT and *n* = 8 amPA biologically independent samples) (**a**) and matching cardiac lysates (**b**). Heat maps show metabolites that are significantly affected by genotype or an interaction between genotype and sex (two-way ANOVA with Tukey’s multiple comparisons test, significance for *P*_adj_: less than 0.05). Differentially abundant (*P*_adj_ < 0.05 for genotype) carnitines and amino acids in plasma (**c**) and cardiac lysates (**d**). Diagonal pattern indicates significant effect of sex (*P*_adj_ < 0.05); hatched pattern indicates significant interaction between sex and genotype (*P*_adj_ < 0.05). Mean ± s.e.m. **e**, Analysis of cardiac acyl-CoA by LC–MS. Heat map summarizes abundance (absolute quantity in pmol mg^−1^ heart tissue) grouped by genotype and sex. Histograms quantify changes, indicating significant (*P* < 0.05) effect of genotype (GEN) or interaction between sex and genotype (SEX×GEN). *n* = 8 amWT male hearts, *n* = 7 amPA male hearts, *n* = 8 amWT female hearts and *n* = 8 amPA female hearts, biologically independent samples. Statistical testing by two-way ANOVA with Tukey’s multiple comparisons test. Mean ± s.e.m. BH(I)B-CoA, β-hydroxy(iso)butyryl-CoA; MM-CoA, methylmalonyl-CoA. */**/*** *P* < 0.05/<0.01/<0.001 for effect of genotype. ^s^/^ss^/^sss^ *P* < 0.05/<0.01/<0.001 for effect of sex. ^#^/^##^ *P* < 0.05/<0.01 for effect of interaction between genotype and sex. F, female; M, male.

Many of the metabolic pathways for propionate involve amino acids either upstream (for example, BCAAs) or downstream (for example, β-alanine/3-aminopropionate). To measure these, we performed metabolomic analyses on paired plasma and cardiac samples using a semi-targeted method that detects derivatised amino acids plus underivatised acyl-carnitines^33^ (Fig. 2a,b). In plasma, 17 metabolites were significantly affected by PA genotype, of which five were also significantly affected by sex (Fig. 2a and Supplementary Table 1). Notable changes included propionyl-carnitine, methionine, aspartate/valine dipeptide (increase) and acetyl-carnitine (decrease) and asparate (decrease in female only, possibly due to aconitase inhibition by 2-methylcitrate)^34^ (Fig. 2c). In cardiac tissue, 137 metabolites were changed in PA, including 22 that were sex dependent and three that showed a significant interaction between sex and genotype (Fig. 2b and Supplementary Table 2). amPA hearts had significantly increased carnitine conjugates of three or more carbons (propionyl, C4:0, C5:0, C6:0 and C7:0; Fig. 2d). An elevation of the carnitine conjugate of succinate/methylmalonate was also detected in amPA, which is somewhat counterintuitive given that these mice have impaired ability to convert propionyl-CoA to methylmalonate by PCC. amPA hearts also had elevated lysine, tyrosine, GABA and various dipeptides (Fig. 2d). Strikingly, only male amPA hearts had raised β-alanine compared to male amWT (Fig. 2d). PA was associated with higher levels of β-alanine dipeptides anserine and carnosine, but the extent of this was greater in males. Overall, the total cardiac β-alanine content was raised in PA but more so in males.

We then performed metabolomic analyses on cardiac lysates to measure short-chain acyl-CoA (Fig. 2e). As expected, propionyl-CoA was greatly elevated in PA hearts, but its excess was larger in female mice. Female PA hearts had a higher excess of isovaleryl-CoA and (iso) butyryl-CoA compared to male PA hearts, which could be a consequence of a bottleneck in BCAA catabolism (isobutyryl-CoA from valine and isovaleryl-CoA from leucine). The PA genotype also associated with higher β-hydroxy β-methylglutaryl-CoA (HMG-CoA), an intermediate of leucine catabolism, as well as ketone and mevalonate pathways. We also observed a decrease in glutaryl-CoA in PA, which could relate to a reduction in lysine metabolism, in agreement with raised lysine levels in PA hearts. Collectively, these data demonstrate that disruption of PCC in PA results in sex-dependent differences in propionate metabolism and acyl-CoA handling.

### Raising β-alanine lessens propionyl-CoA buildup

We found that the metabolic disturbance associated with propionyl-CoA is less profound in male PA hearts and associates with a higher buildup of β-alanine. This non-proteinogenic amino acid can be synthesised from propionyl-CoA and is the rate-limiting precursor of carnosine and anserine—dipeptides with well-established antioxidant^35^ and pH/Ca^2+^-buffering^36^ properties in the heart^28^. The lower propionyl-CoA excess in male PA hearts may be explained by augmented production capacity of carnosine; indeed, overexpression of carnosine synthase in mice reduced cardiac propionate levels^35^. Higher levels of β-alanine-containing dipeptides would, in turn, facilitate mitochondrial function and metabolic flows. If β-alanine conferred such protection, then raising its bioavailability in propionate-stressed cells in vitro should reduce the propionyl-CoA excess. If, in contrast, β-alanine were merely an ‘endpoint’ for propionyl-CoA metabolism, then raising intracellular β-alanine experimentally would cause propionyl-CoA to build up.

**Fig. 3:**
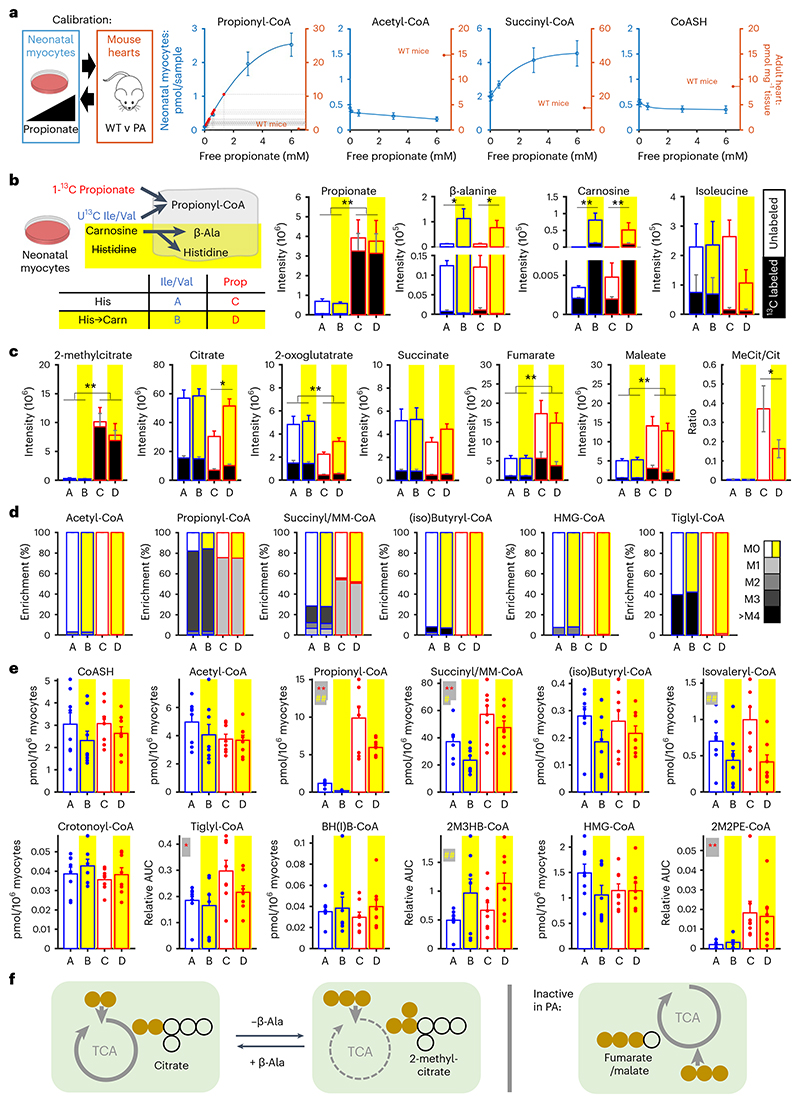
Raising and lessening propionyl-CoA in vitro with medium interventions. **a**, Calibration experiment to titrate the amount of exogenous propionate needed produce a rise in propionyl-CoA in the range found in PA hearts. Forty-eight-hour treatment of NRVMs with up to 6 mM sodium propionate. Measurements of acyl-CoAs in NRVM lysates (*n* = 6 biologically independent samples from six isolations; left axis) were compared to measurements in PA and WT mice (right axis). Mathematical minimisation procedure best-fitted calibration constants to indicate that ~1 mM propionate produces a rise in propionyl-CoA within the range of PA mice (red dots). Mean ± s.e.m. **b**, ^13^C tracing experiment in NRVMs with raised propionyl-CoA (exogenous propionate treatment) and for raised β-alanine (histidine in medium replaced with carnosine; yellow banding). Table shows the four treatment conditions (A/B/C/D). For conditions A/B, Ile and Val were uniformly labeled (^13^C on all carbons). For conditions C/D, 1 mM labeled propionate (^13^C at carbon-1) was added to the medium. Condition C/D produced a rise in propionate. *n* = 7 biologically independent samples per condition from seven isolations. Mean ± s.e.m. */** *P* < 0.05/<0.01. **c**, IC–MS identifies Krebs cycle intermediates. MeCit/Cit, 2-methylcitrate/citrate ratio. */** *P* < 0.05/<0.01. Mean ± s.e.m. **d**, ^13^C tracing of Ile/Val carbons (conditions A/B) or propionate carbon-1 (conditions C/D), expressed as percent molar enrichment. *n* = 7 per condition from seven isolations. Key shows number of ^13^C-labeled atoms. **e**, Acyl-CoA measurements expressed either as absolute quantification calibrated against standard curves or as relative AUC (without standards). *n* = 7 biologically independent samples per condition from seven isolations. */** *P* < 0.05/<0.01 for effect of propionate treatment. ^#^/^##^ *P* < 0.05/<0.01 for effect of raising β-alanine. Statistical testing by repeated-measures two-way ANOVA. Mean ± s.e.m. **f**, Summary of findings. Brown-filled symbols indicate carbons of acetyl-CoA or propionyl-CoA competing for entry into the Krebs cycle as citrate or 2-methylcitrate. Raising β-alanine favors the former. Propionate also enters the Krebs cycle as succinyl-CoA, but this pathway is not normally available in PA due to PCC inactivation. 2M2PE-CoA, *trans*-2-methyl-pentenoyl-CoA; 2M3HB-CoA, 2-methyl-3-hydroxybutyryl-CoA; BH(I)B-CoA, β-hydroxy(iso)butyryl-CoA; Ile, isoleucine; MM-CoA, methylmalonyl-CoA; Val, valine.

We tested these models by ^13^C tracing experiments in cultured WT neonatal rat ventricular myocytes (NRVMs). To determine the concentration of propionate required to raise propionyl-CoA to the range in PA hearts in vivo, acyl-CoAs were quantified as a function of exogenous propionate concentration (Fig. 3a). We determined that 1 mM propionate in vitro matched the mid-range of propionyl-CoA in PA hearts. To trace propionate metabolism, exogenous 1-^13^C-propionate was used. To trace the metabolism of propiogenic substrates (without exogenous propionate), isoleucine and valine were replaced with ^13^C-labeled equivalents. To load cells with β-alanine, we replaced histidine, an essential amino acid, with carnosine, which forces myocytes to sequester β-alanine. Thus, four conditions were studied: medium with labeled BCAAs (A), medium with labeled BCAAs and histidine replaced with carnosine (B), medium with labeled propionate (C) and medium with labeled propionate and histidine replaced with carnosine (D) (Fig. 3b). We identified a significant rise in labeled isoleucine in conditions A and B and a rise in labeled propionate in conditions C and D, as well as the emergence of carnosine and β-alanine in conditions B and D, indicating that the loading maneuver was successful (Fig. 3b). Some carnosine was ^13^C labeled, indicating that propiogenic substrates or propionate are converted to this dipeptide. The increase in propionate concentration caused a buildup of 2-methylcitrate and a reduction in citrate, indicating Krebs cycle inhibition (Fig. 3c). However, β-alanine restored citrate and halved the 2-methylcitrate/citrate ratio^37^ (Fig. 3c). Of note, propionate also increased levels of labeled and unlabeled fumarate and malate, indicating recruitment into the Krebs cycle via succinyl-CoA, although this pathway would be inactive in PA.

Additional ^13^C tracing experiments confirmed the incorporation of propionate into propionyl-CoA (at carbon-1) and the metabolic processing of BCAA, as shown by the rise in labeled tiglyl-CoA (isoleucine metabolite) and (iso)butyryl-CoA (valine metabolite) and, eventually, propionyl-CoA (at all carbons) (Fig. 3d). Loading cells with β-alanine reduced propionyl-CoA levels (B versus A and D versus C; Fig. 3e). We found that propionate hinders leucine metabolism by raising isovaleryl-CoA and tiglyl-CoA levels, but this effect was less-ened with raising β-alanine. These observations suggest that raising β-alanine facilitates metabolic flows related to propionyl-CoA (Fig. 3f).

### Disturbed propionyl-CoA metabolism affects contraction

**Extended Data Fig. 1:**
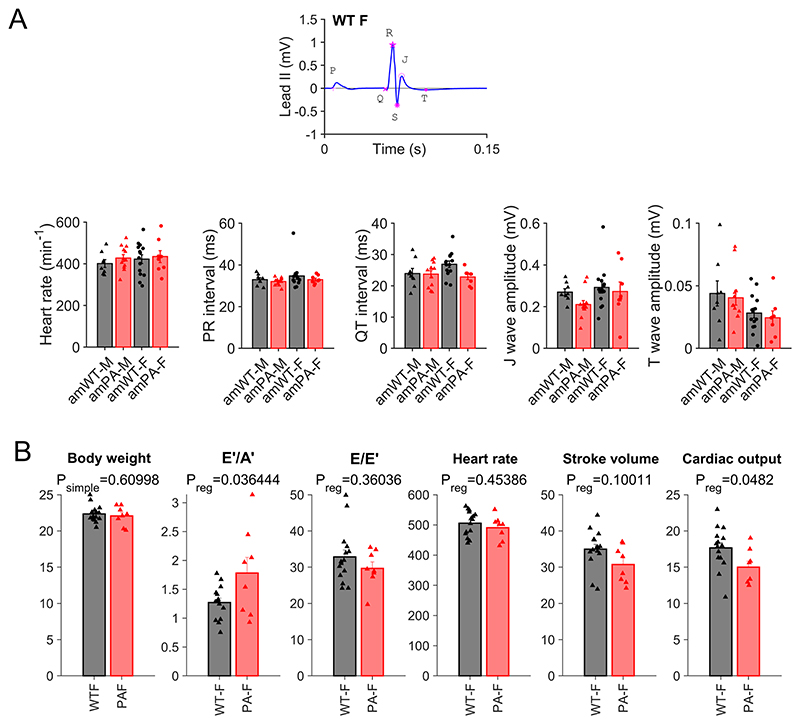
*In vivo* cardiac phenotyping of mice with disrupted propionyl-CoA handling. **(a)** Exemplar electrocardiogram recorded in lead II, showing waves and intervals, in 8-week-old amPA or amWT mice. Heart rate, intervals and wave amplitudes (N = 8, 12, 14, 8). Two-way ANOVA with Tukey’s multiple comparisons test. Mean ± SEM. **(b)** Echocardiography of female mice scanned at 8 weeks of age. Body weight of mice used for echocardiography, E/E’ and E’/A’ ratio measured from pulsed-wave and Tissue Doppler in apical four-chamber view; stroke volume and cardiac output measured from M-mode scans in the parasternal short-axis view (N = 15, 8). Significance tested by regression analysis. Mean ± SEM.

**Fig. 4:**
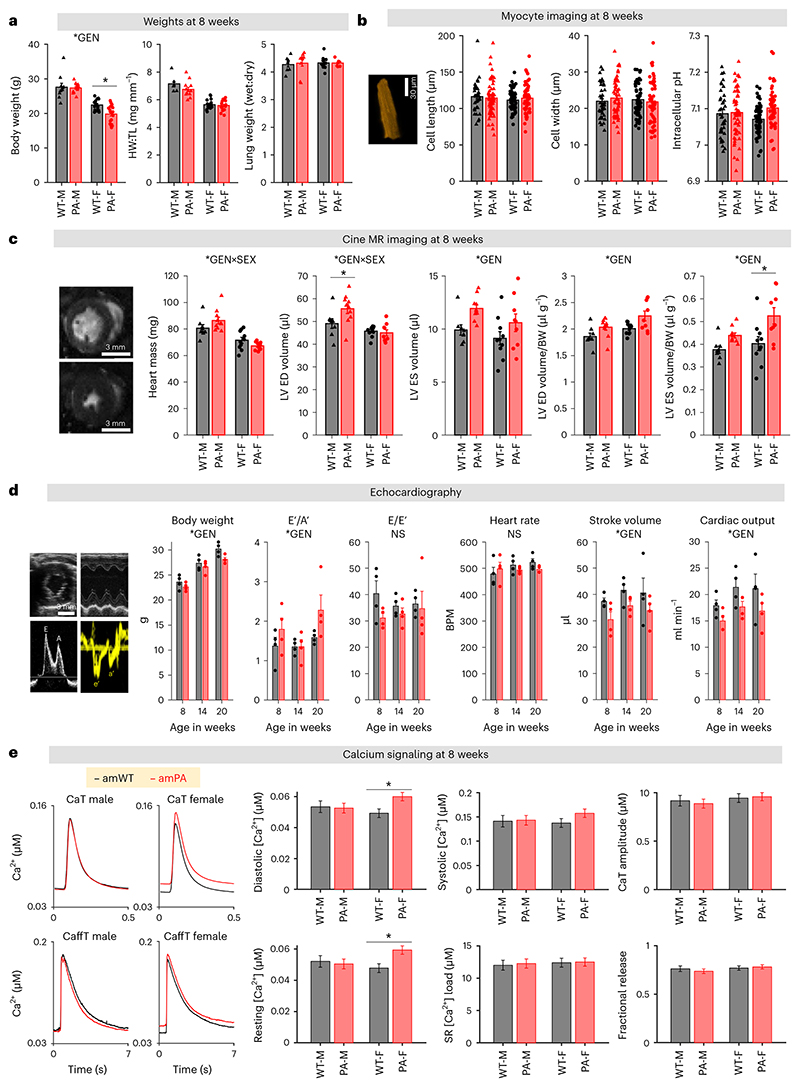
Mice with disrupted propionyl-CoA handling develop cardiac contractile dysfunction. **a**, Body weight, heart weight (HW):tibia length (TL) ratio and wet:dry lung weight ratio (*n* = 10, 14, 16 and 19 animals). *GEN denotes significant (*P* < 0.05) effect of genotype. Two-way ANOVA followed by multiple comparisons test. **b**, Cell dimensions and intracellular pH measured in cSNARF1-loaded myocytes. Hierarchical analyses from a total of 93–113 cells per genotype from 4–5 isolations. **c**, Cine MR imaging of 8-week mouse hearts showing rendered heart mass, LV end-diastolic (ED) end-systolic (SV) volumes, and LVED and LVES after normalising to body weight—that is, indexed (*n* = 8, 10, 10 and 9 animals). *GEN denotes significant (*P* < 0.05) effect of genotype. *GEN×SEX denotes significant (*P* < 0.05) interaction between sex and genotype (ordinary two-way ANOVA with Tukey’s multiple comparisons test). **d**, Echocardiography of female mice scanned at three timepoints (8 weeks, 14 weeks and 20 weeks) and their body weight. E/E′ and E′/A′ were measured from pulsed wave and tissue Doppler in apical four-chamber view; SV and cardiac output were measured in parasternal short-axis view (*n* = 4 and *n* = 4 animals, repeated-measures ANOVA). *GEN denotes significant (*P* < 0.05) effect of genotype. Mean ± s.e.m. **e**, Calcium signaling measured by fluorescence imaging of myocytes freshly isolated from 8-week-old amPA or amWT hearts. Protocol measured electrically evoked CaTs to obtain diastolic and systolic Ca^2+^ and CaT amplitude (amp). After a train of CaTs, a CaffT was produced to interrogate resting Ca^2+^, SR Ca^2+^ load and fractional release. Hierarchical analysis of 226–240 myocytes from *n* = 7, 7, 8 and 9 isolations. *P < 0.05. Mean ± s.e.m. BPM, beats per minute; BW, body weight; F, female; M, male; NS, not significant.

The functional impact of the PA-linked metabolic disturbance was studied in terms of cardiac contraction. In particular, we investigated whether the sex dependence in metabolic changes also produces a sex-dependent cardiac phenotype. At 8 weeks, body weight was significantly lower in female (but not male) amPA. There was no evidence for cardiac hypertrophy (heart weight:tibia length and cell size) or pulmonary congestion (lung wet:dry weight) (Fig. 4a,b). Although PA is a form of systemic acidosis, intracellular pH (pH_i_) in myocytes was unchanged (Fig. 4b). We found no major changes in the electrocardiogram (Extended Data Fig. 1a and Supplementary Table 3), but cine magnetic resonance (MR) imaging revealed that end-systolic volume (ESV), controlled for body weight, was more profoundly increased in female PA hearts, indicative of a less complete ejection (Fig. 4c and Supplementary Table 4). This was verified by echocardiography, which showed reduced cardiac output at 8 weeks (Extended Data Fig. 1b and Supplementary Table 5), persisting at 14 weeks and 20 weeks in a subset of animals selected for a longitudinal study (Fig. 4d). Although an increase in E′/A′ was detected on tissue Doppler imaging, this was not associated with a change in E/E′, the clinical marker of diastolic function.

**Extended Data Fig. 2:**
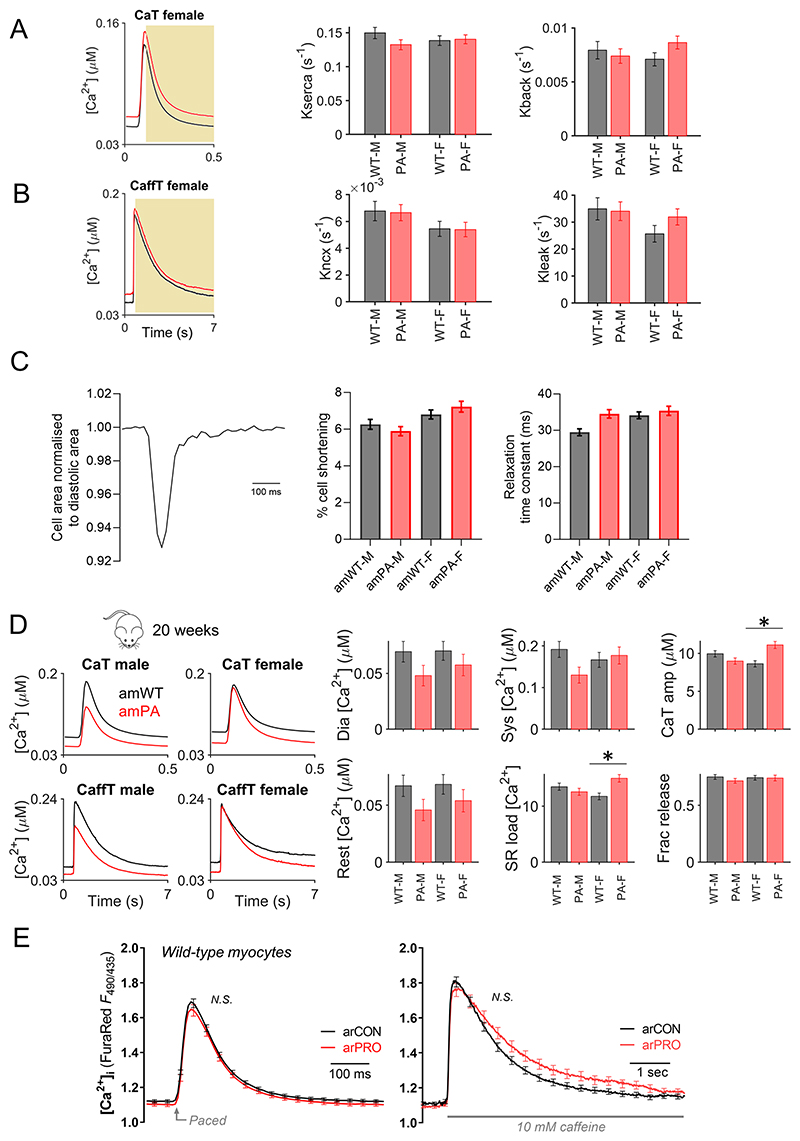
Ca^2+^ handling and cell-shortening analysis in 8-week-old and 20-week-old adult mouse ventricular myocytes loaded with FuraRed. Analysis of **(a)** SERCA and **(b)** NCX function, and the fluxes needed to balance their activity (SR backflux and sarcolemmal leak, respectively), from electrically-evoked Ca^2+^ transients (CaT) and caffeine-evoked Ca^2+^ release (CaffT) in myocytes isolated from mice at 8 weeks of age. SERCA pump activity was described in terms of a rate constant (unit: s^−1^) which was calculated from the slope of the relationship between flux and cytoplasmic [Ca^2+^]. To produce this relationship, flux was calculated from the recovery phase of the CaT, and multiplied by buffering capacity to give d[Ca^2+^]/dt. Backflux was calculated as the rate required to balance SERCA at diastolic [Ca^2+^]. The analysis for NCX used the recovery from caffeine-evoked Ca^2+^ release. Sarcolemmal leak was calculated as the rate required to balance NCX at resting [Ca^2+^]. Time-course shown as mean only. Hierarchical analysis of 226–240 myocytes from N = 7, 7, 8, 9 isolations. Mean ± SEM. **(c)** Hierarchical analysis of cell-shortening of myocytes from 8 week mice, obtained from the cell area during electrically-evoked CaT. Mean ± SEM. **(d)** Analysis of electrically evoked Ca^2+^ transients and caffeine evoked Ca^2+^ release in myocytes isolated from mice at 20 weeks of age. See Fig. 3 for details of methods. Hierarchical analysis of results from 4-5 isolations per sex and genotype, yielding 25–37 myocytes per group. * P < 0.05. Mean ± SEM. **(e)** Recordings of electrically-evoked Ca^2+^ transients and caffeine-evoked Ca^2+^ release made in adult rat (ar) ventricular myocytes exposed for 10–30 mins to control (CON) or 6 mM propionate (PRO). Time-course shown as mean ± SEM. Hierarchical analysis of 54-67 myocytes from N = 5 isolations (N.S; not significant).

Less complete ejection is consistent with a higher ESV and lower stroke volume (SV), and one possible mechanism is dysregulated Ca^2+^ signaling. We imaged Ca^2+^ in isolated myocytes loaded with the ratiometric, red-shifted Ca^2+^indicator FuraRed. A ratiometric readout is critical for enabling comparisons between groups^38^, and red-shifted fluorescence is less susceptible to bleed-through from mitochondrial autofluorescence that may be altered in metabolic disorders. A limitation of FuraRed is its low Ca^2+^ dissociation constant (K_d_), which gives weaker resolving power at higher Ca^2+^. However, the FuraRed calibration curve^39^ indicates saturation for ratios greater than 2.4, which is sufficient to provide good resolving power at the peak of Ca^2+^ events. Ca^2+^ handling was interrogated using a concatenated protocol^39^ that consisted of a train of electrically evoked calcium transients (CaTs), followed by a caffeine-evoked Ca^2+^ release (CaffT) from the sarcoplasmic reticulum (SR) (Fig. 4e). Recovery from the CaT and CaffT informed the SR Ca^2+^ ATPase (SERCA) activity (Extended Data Fig. 2a) and sarcolemmal Na^+^/Ca^2+^ exchange (NCX) activity (Extended Data Fig. 2b), respectively. Given the lack of evidence for cellular hypertrophy, cytoplasmic Ca^2+^ buffering capacity was assumed to be no different in amPA. At 8 weeks, we observed a rise in diastolic [Ca^2+^] in female amPA myocytes (Fig. 4e). This occurred without a rise in systolic [Ca^2+^], which is striking because, normally, higher diastolic [Ca^2+^] leads to greater SR Ca^2+^ loading and larger CaTs^38^. Overall, CaT amplitude was not significantly affected, which argues against systolic dysfunction, as supported by measurements of cell-shortening in isolated myocytes (Extended Data Fig. 2c). Higher diastolic [Ca^2+^] implies a right-shift in the [Ca^2+^]-activation curve of SERCA—for example, due to a reduction in apparent Ca^2+^ affinity. The steepness of SERCA’s [Ca^2+^]-flux relationship was, however, unaltered in PA (Extended Data Fig. 2a,b). Thus, at matching [Ca^2+^] SERCA flux was attenuated in female amPA myocytes, which explains why the higher diastolic Ca^2+^ did not evoke a larger CaT. Higher diastolic [Ca^2+^] leads to less complete myofilament deactivation, particularly at the physiological heartrate of mice. Such diastolic dysfunction would explain the lower SV and higher ESV observed in PA mice. The evolution of changes in CaTs was followed to 20 weeks, at which additional stress and compensatory responses may affect the heart. In older adults, female PA mice had more substantial remodeling, characterised by an increase in CaT amplitude, possibly reflecting compensation (Extended Data Fig. 2d).

**Extended Data Fig. 3:**
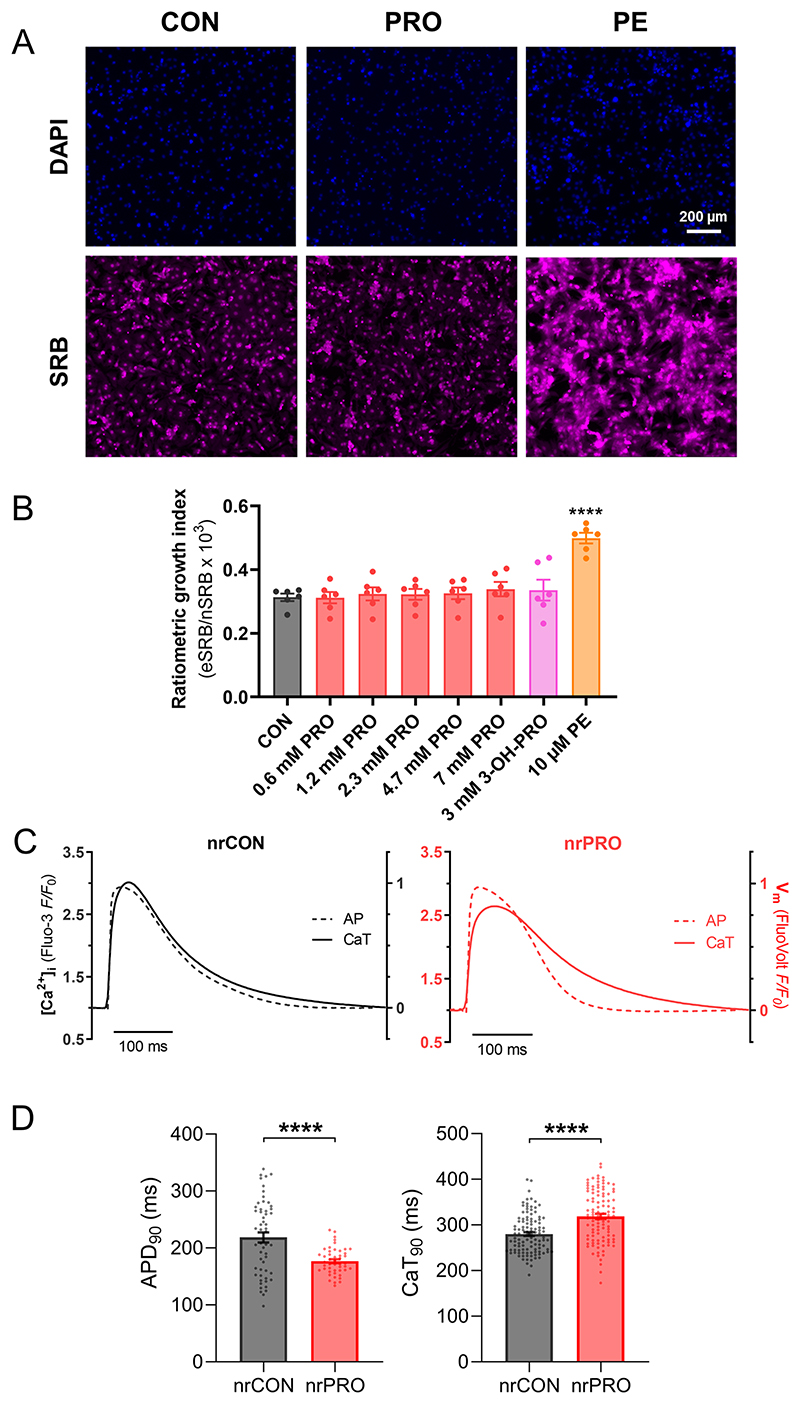
Treatment of myocytes with propionate *in vitro* remodels the coupling of action potentials and Ca^2+^ transients in the absence of hypertrophy. **(a)** Sulforhodamine B (SRB) staining of NRVMs treated with propionate (PRO), its 3-hydroxylated derivative (3-OH-PRO) or phenylephrine (PE; pro-hypertrophic agonist) for 48-h. (A) Exemplar images of DAPI and SRB co-staining in control (CON), PRO (4.7 mM) and PE. **(b)** Ratiometric SRB growth index (eSRB, extra-nuclear SRB signal; nSRB, nuclear SRB signal). N = 6 biologically independent measurements from 6 isolations, with each measurement determined from technical quadruplicates. Ordinary one-way ANOVA with Dunnett’s multiple comparisons test. **** P < 0.0001. Mean ± SEM. **(c)** Fluorescence imaging of wild-type rat myocytes treated with exogenous propionate. Averaged time-courses of electrically-evoked action potentials (AP; dotted line) and Ca^2+^ transients (CaT; solid line) made in FluoVolt or Fluo3-loaded neonatal rat (nr) ventricular myocytes respectively, treated for 48-h under control (CON) or 6 mM propionate (PRO) conditions under steady-state pacing at 2 Hz. Time-courses shown as mean only (N = 47–58 myocytes from 7 isolations for FluoVolt, and N = 104–109 myocytes from 9 isolations for Fluo-3). **(d)** Action potential duration at 90% repolarisation (APD_90_) and time to 90% CaT recovery (CaT_90_). Hierarchical analysis of N = 47–58 myocytes from 7 isolations for FluoVolt, and N = 104-109 myocytes from 9 isolations for Fluo-3 shown as mean ± SEM. Two-sided, two sample T-test corrected for hierarchical analysis. **** P < 0.0001.

To test if the presence of propionate anions affects Ca^2+^ handling acutely, CaTs were measured in WT adult rat myocytes superfused with propionate-containing solution. Propionate did not affect Ca^2+^ signals (Extended Data Fig. 2e). To test for slower-onset actions, WT NRVMs were cultured in the presence of 6 mM propionate, shown earlier to produce a saturating increase in propionyl-CoA (Fig. 3a). We saw no hypertrophic effect (Extended Data Fig. 3a,b) but a profound lengthening of the CaT, relative to its action potential (Extended Data Fig. 3c,d). These findings indicate a possible transcriptional effect of propionate.

### Transcriptional changes evoked by propionate/propionyl-CoA

**Extended Data Fig. 4:**
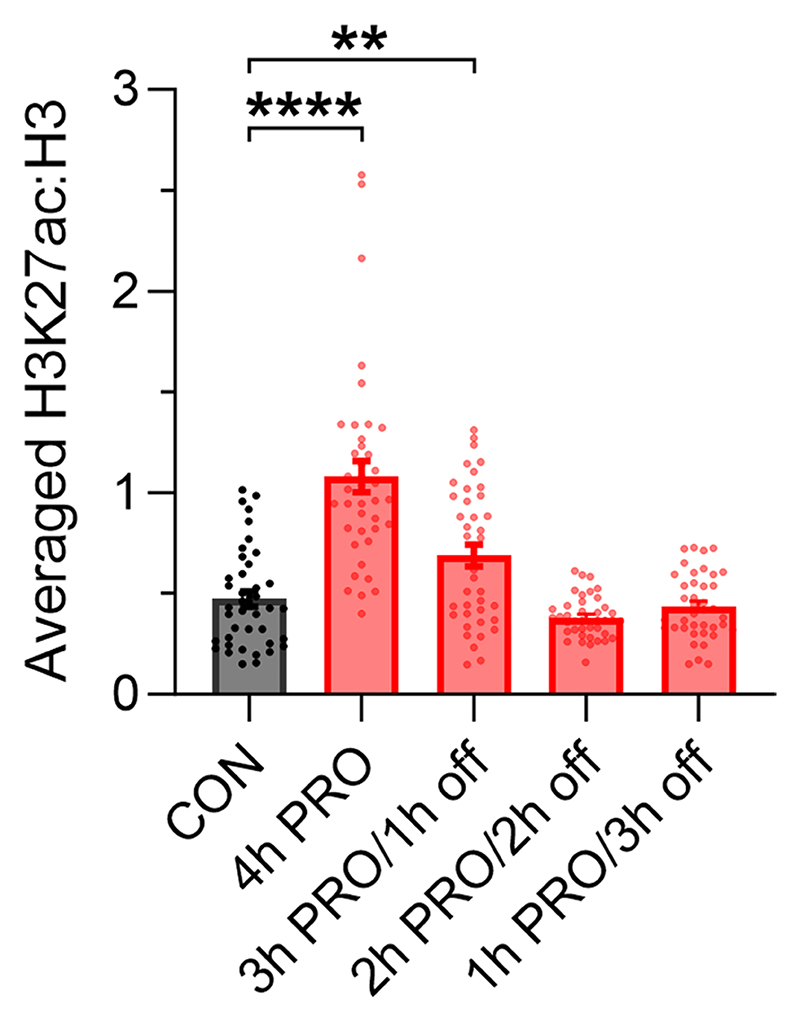
Propionate-dependent increases in nuclear histone H3 lysine 27 acetylation are temporally labile. Propionate-evoked increase in H3K27ac studied by immunofluorescence imaging of PRO-treated adult myocytes following a 4-h time-course; ‘off’ indicates replacement of PRO with CON media. Data shown as H3K27ac:H3 signal averaged over the nucleus. N = 40 nuclei (from 20 adult myocytes) from 2 isolations. Ordinary one-way ANOVA with Dunnett’s multiple comparisons test. **/**** P < 0.01/0.0001. Mean ± SEM.

**Extended Data Fig. 5:**
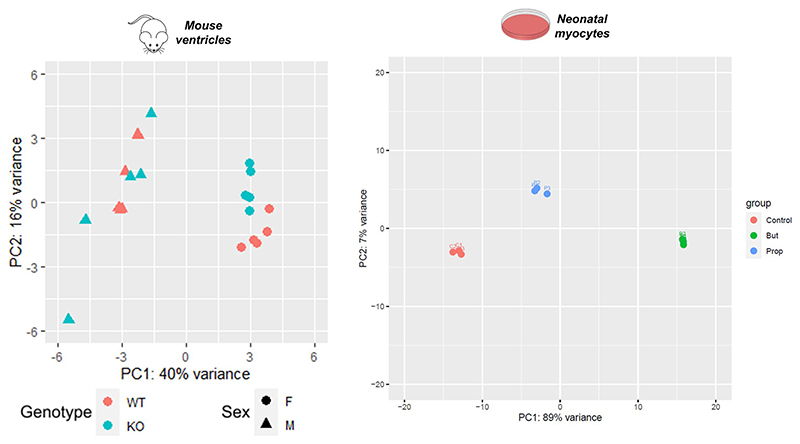
Principal component analysis of transcriptomic data. Principal component analysis of transcriptomic data from PA (‘KO’) and WT mice (F-female, M-male) (N = 5, 5, 5, 5) and in NRVMs treated with control media or media supplemented with propionate (Prop) or butyrate (But) (N = 3, 3, 3).

**Extended Data Fig. 6:**
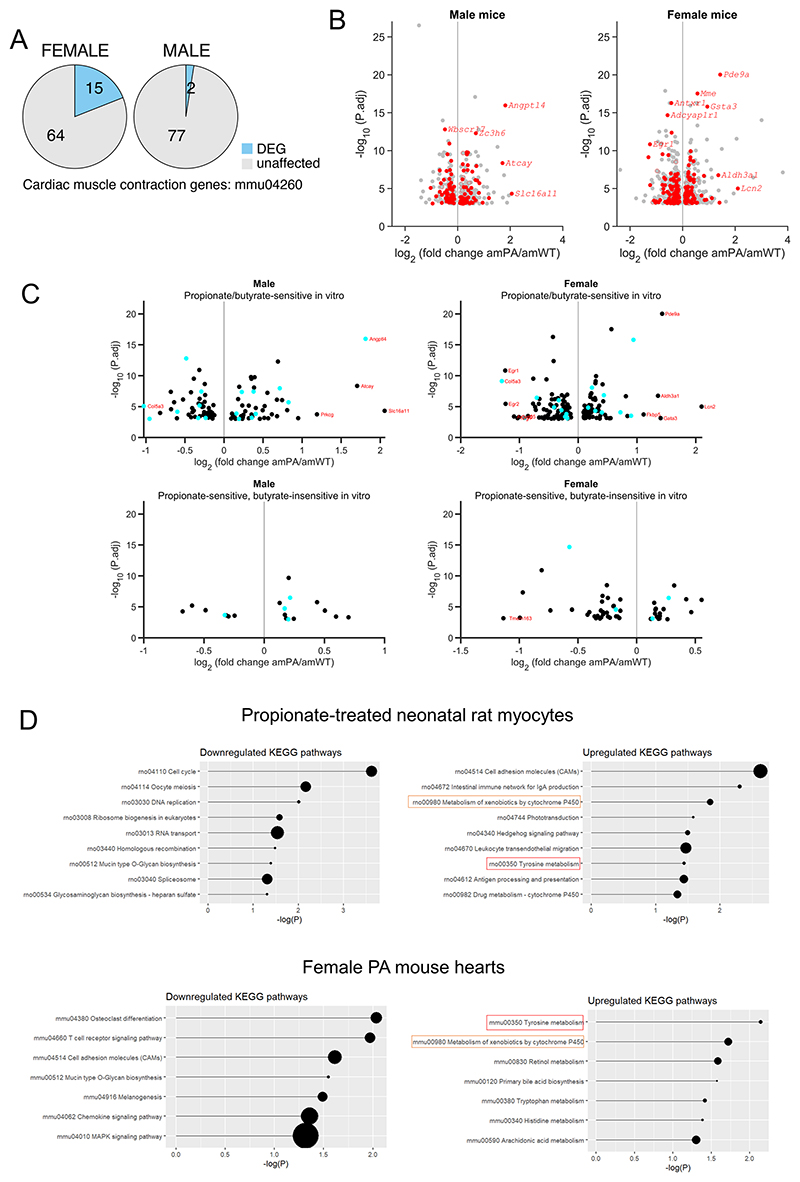
Analysis of differentially expressed genes from transcriptomic analysis. **(a)** Number of genes in the mmu03260 KEGG pathway that are differentially expressed in female and male PA mice, relative to wild-type. **(b)** DEGs identified in female and male amPA, relative to amWT. Red filled symbols identify DEGs found also to be propionate- and butyrate-sensitive *in vitro*. Red empty symbols identify DEGs that were also found to be sensitive to propionate, but not butyrate, *in vitro*. The remaining DEGs, appearing as grey symbols, were identified in mice only, but not *in vitro*. **(c)** Volcano plots for DEGs identified in male or female mice, categorized by sensitivity to propionate/butyrate in cultured neonatal rat ventricular myocytes. DEGs in light blue were identified in both sexes. Labeled DEGs are genes of interest. **(d)** Lollipop graph of KEGG pathway analysis from DEGs identified in female PA mice and propionate-treated NRVMs. Red box indicates common enrichments. Adjusted P < 0.05.

**Fig. 5:**
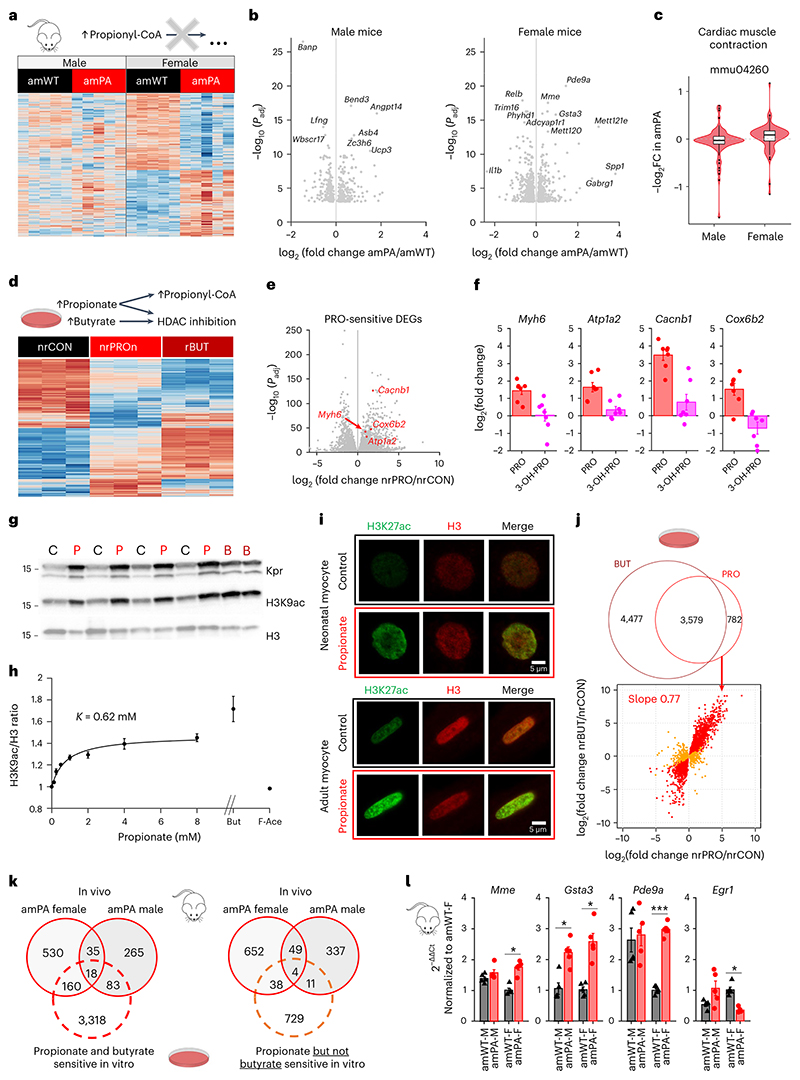
Cardiac gene expression changes in response to propionate. **a**, RNA-seq analysis of cardiac lysates from sex-balanced amPA and amWT hearts (*n* = 5 biologically independent samples each). Heat map shows all genes significantly affected by genotype (*P*_adj_ < 0.05), grouped by sex. DESeq2 analysis using design ~genotype+sex+sex:genotype. **b**, Volcano plot showing DEGs identified in male and female amPA, relative to amWT. Selected genes are labeled. Corrected for multiple comparisons. **c**, Violin plot of the log_2_(fold change (FC)) in expression of genes of the cardiac muscle contraction KEGG pathway in male and female PA mice, relative to WT. Midline shows median, with upper and lower hinges showing 25th and 75th percentiles, respectively. Upper and lower whiskers extend to the largest and smallest data points within 1.5 times the interquartile range of either hinge. **d**, RNA-seq analysis of lysates obtained from cultured NRVMs treated with 6 mM propionate (nrPRO) or 6 mM butyrate (nrBUT) for 24 h. Heat map shows responses, relative to untreated controls (nrCON). Experiments were grouped from three biologically independent isolations. **e**, Volcano plot shows transcriptional responses to propionate treatment, highlighting four genes of ‘cardiac muscle contraction’ pathway. Corrected for multiple comparisons. **f**, RT–qPCR validation of selected genes confirms effect of propionate but not of 3-hydroxy derivative (*n* = 6 per condition from four isolations). Paired two-tailed *t*-test. Mean ± s.e.m. **g**, Western blot of histone extracts from cultured neonatal myocytes treated with 3 mM propionate (P), 3 mM butyrate (B) or control (C), probed using antibodies against pan-propionylation (Kpr), H3K9ac and total histone H3. Includes repeats from independently collected lysates (*n* = 4 C, 4 P and 2 B from four isolations). **h**, Whole-cell ELISA measurements of H3K9ac-to-H3 ratio in fixed neonatal myocytes treated for 24 h, normalized to untreated control. Best-fit (non-cooperative Hill curve) indicates half-maximal concentration (*n* = 6 biologically independent measurements from six isolations, with each measurement determined from technical triplicates). Mean ± s.e.m. But, 3 mM butyrate; F-Ace, 3 mM 2-fluoroacetate. **i**, Immunofluorescence imaging of adult and neonatal myocyte nuclei after treatment (4 h or 24 h, respectively) with propionate, showing H3K27ac response. Further quantification in Extended Data Fig. 7. **j**, Comparison of DEGs sensitive to propionate and butyrate in vitro. Red symbols denote DEGs that respond to both propionate and butyrate (Pearson’s *r* = 0.9198); orange symbols denote DEGs that respond to propionate only (Pearson’s *r* = −0.1456). **k**, Venn diagrams showing number of DEGs according to response in vivo (mouse heart) and in vitro (cultured myocytes) grouped by sex. **l**, RT–qPCR of selected propionate-sensitive DEGs in mouse hearts (*n* = 5, 5, 5 and 5 biologically independent samples). Ordinary two-way ANOVA with Tukey’s multiple comparisons test. */*** *P* < 0.05/<0.001. Mean ± s.e.m. F, female; M, male.

We performed RNA sequencing (RNA-seq) analysis on snap-frozen ventricular tissue from 8-week amWT and amPA mice to seek transcriptional responses. Lysates were prepared from ventricular tissue rather than isolated myocytes because of concerns that the relatively long digestion procedure may affect propionate-dependent epigenetic changes, which can be short-lived (Extended Data Fig. 4). Principal component analysis demonstrated a separation by genotype in female mice (Extended Data Fig. 5). We identified more than 1,000 differentially expressed genes (DEGs) in PA mice (Fig. 5a,b; *P*_adj_ < 0.05), most of which were sex specific (690 in females, 348 in males), with only 53 concordant responses. Female PA mice showed a more profound transcriptional response, including a significant upregulation of genes in the ‘cardiac muscle contraction’ pathway (*P*_adj_ = 10^−4^; Fig. 5c and Extended Data Fig. 6a–c).

To define the gene set that responds directly to propionate/propionyl-CoA, we measured short-term (24-h) transcriptional responses to propionate in NRVMs that lack confounding systemic influences. To detect the widest scope of ‘propionate-sensitive’ DEGs, we treated myocytes with 6 mM propionate to evoke a saturating rise in propionyl-CoA (Fig. 3a). The in vivo propionate response is likely to be much milder; thus, only a subset of in vitro DEGs is expected to overlap with DEGs identified in PA mice. In vivo DEGs that are not detected in vitro are unlikely to be a primary response to propionate/propionyl-CoA but possibly a stress or compensatory response instead. Propionate applied to NRVMs (nrPRO) resulted four times as many DEGs as in amPA mice (Fig. 5d,e). KEGG pathway enrichment analyses identified several amino acid and toxin-handling pathways as upregulated in PA mice, of which ‘tyrosine metabolism’ and ‘xenobiotic metabolism by P450’ were also detected in nrPRO (Extended Data Fig. 6d). To test how the in vitro responses relate to propionate chemistry, we treated NRVMs with 3-hydroxypropionate but found that selected genes were not induced (RT–qPCR; Fig. 5f). Thus, other polar derivatives detected by metabolomics (for example, 3-acetylpropionate) are unlikely to evoke transcriptional responses.

**Extended Data Fig. 7:**
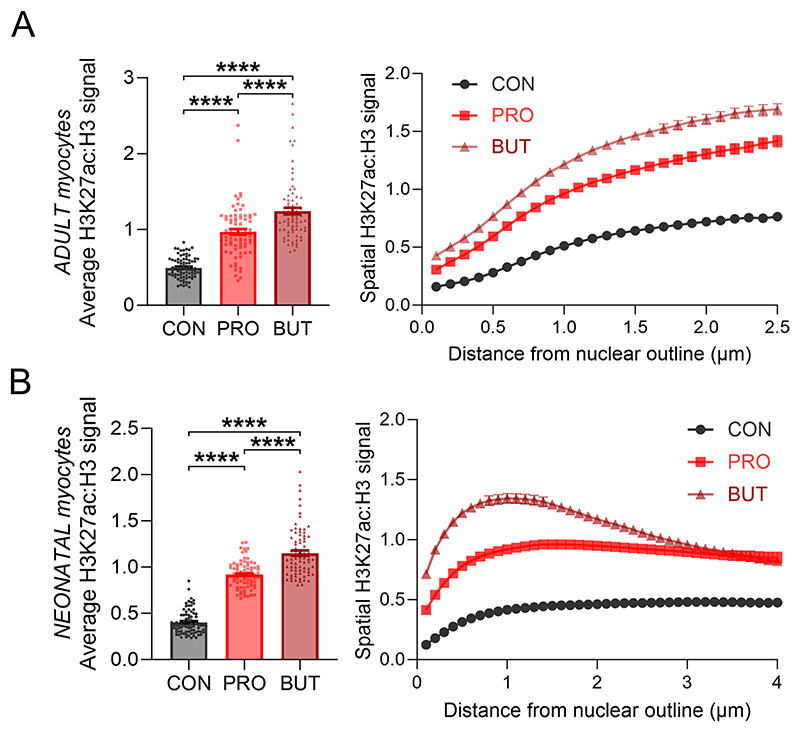
Histone H3 lysine 27 acetylation is increased in a spatially consistent manner in the nuclei of myocytes treated with propionate *in vitro*. H3K27ac and H3 immunofluorescence imaging of adult and neonatal myocyte nuclei following 4-h and 24-h treatment respectively, with 6 mM propionate (PRO) or 6 mM butyrate (BUT). Summary data for H3K27ac in **(a)** adult and **(b)** neonatal myocytes. Data shown as the average H3K27ac:H3 signal averaged over the nucleus *(left)* and the H3K27ac:H3 ratio plotted against distance from the nuclear outline (μm) *(right)*. Hierarchical analysis of 80 nuclei (from 40 adult myocytes) and 80 nuclei (from 80 neonatal myocytes) from N = 4 isolations. Ordinary one-way ANOVA with Tukey’s multiple comparisons test. **** P < 0.0001. Mean ± SEM.

Propionate treatment could increase histone acylation as a substrate for direct propionylation by acyl-transferases^9^or by increasing net histone acetylation, either through HDAC inhibition^8,14,15^ or by activating p300 acetyl-transferase^13^. To parse these effects, NRVMs were treated with butyrate, a 4-carbon acyl that also inhibits HDACs and activates p300. Both propionate and butyrate induced a significant increase in acetylation of H3K9ac (Fig. 5g). Propionate, additionally, increased histone propionylation, which was detected with a pan-propionyllysine (Kpr) antibody (Fig. 5g). Propionate’s half-maximal effect on H3K9ac was at ~0.6 mM and saturated at approximately 80% of the response to butyrate (Fig. 5h). Interestingly, 2-fluoroacetate, which previous studies have shown to produce propionate-like toxicity^40^, did not affect H3K9ac levels. Treatment with propionate increased H3K27ac signal in nuclei, although neonatal myocytes showed a more peripheral effect compared to adult myocytes (Fig. 5i and Extended Data Fig. 7). Our data indicate that propionate/propionyl-CoA can act as acyl-transferase substrate for propionylation and also alter net histone acetylation in a manner similar to butyrate. To identify the genes in the latter group, the transcriptional response of nrPRO (4,361 DEGs) was compared to responses to in vitro butyrate treatment (nrBUT; 8,056 DEGs; Fig. 5j). Eighty-two percent of the propionate-sensitive DEGs also responded to butyrate and produced a strong correlation, presumably reflecting their common denominator of increased acetylation. The remaining propionate-sensitive DEGs (782, 18%) did not correlate positively with butyrate and are likely to reflect propionate-specific actions, including propionylation.

**Extended Data Fig. 8:**
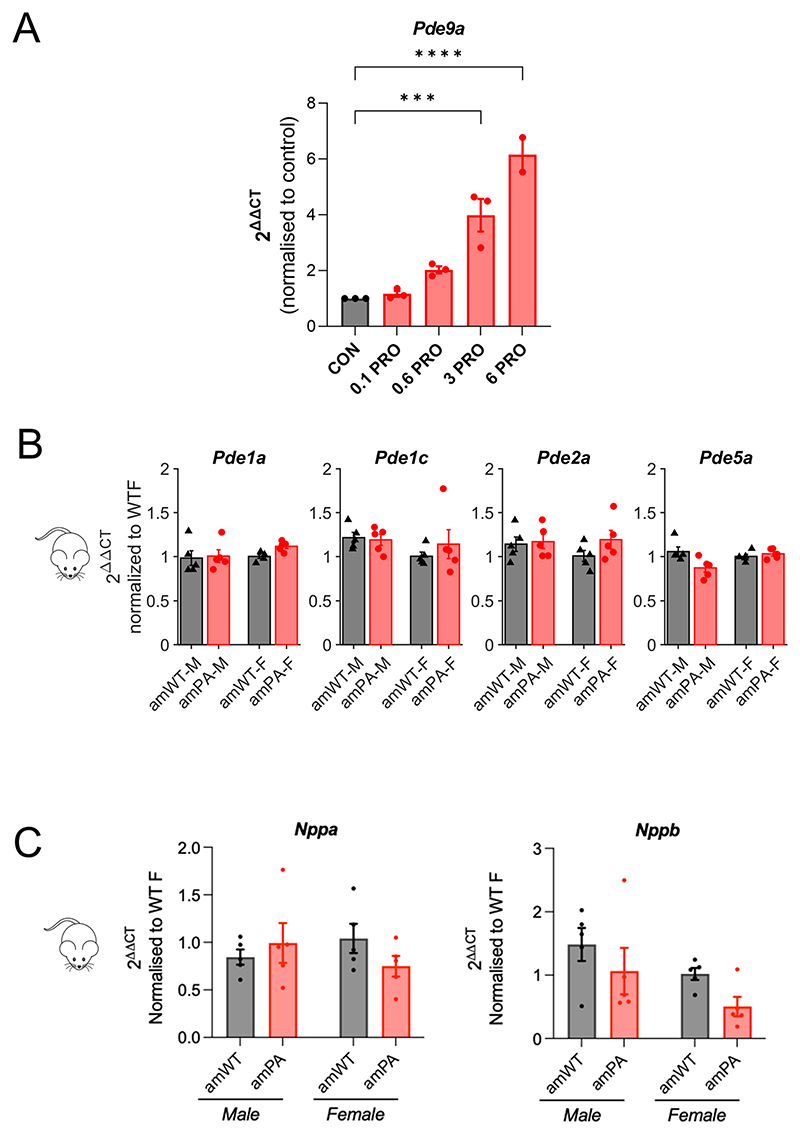
Transcriptional responses to propionate *in vitro* and disrupted propionyl-CoA handling *in vivo*. **(a)** Concentration-dependence of *Pde9a* induction following 24-h exogenous propionate treatment in NRVMs *in vitro*. Shown are 2^ΔΔCT^ values normalised to control (CON). Results from N = 4 isolations per condition. Ordinary one-way ANOVA with Dunnett’s multiple comparisons test. ***/**** P < 0.001/0.0001. Mean ± SEM. **(b)** RT-qPCR of adult mouse heart lysates showing lack of effect of PA or sex on the expression of major PDE-coding genes (except *Pde9a*) or **(c)** in natriuretic peptide A and B expression (N = 5, 5, 5, 5). Mean ± SEM.

Finally, we compared nrPRO transcriptional responses with DEGs identified in PA mice. In total, 261 DEGs were identified as both PA responsive in vivo and sensitive to both propionate and butyrate in vitro. Of these, most (178) were identified in females, with only 18 in both sexes (Fig. 5k). These DEGs can be attributed to a direct effect of propionate/propionyl-CoA in the amPA heart (Extended Data Fig. 6b,c). Examples of prominently affected genes in vivo and in vitro included *Pde9a, Mme, Gsta3* and *Egr1. Pde9a* induction in NRVMs was concentration-dependent with [propionate], having a half-maximal effect near ~1 mM propionate (Extended Data Fig. 8a). Transcript levels (RT–qPCR) confirmed *Egr1* downregulation and *Gsta3* upregulation in both male and female PA mice and that the upregulation of *Pde9a* and *Mme* was specific to females. Intriguingly, WT *Mme* and *Pde9a* transcript levels were higher in male mice (Fig. 5l).

### Changes in cyclic nucleotide signaling and protein phosphorylation

**Fig. 6:**
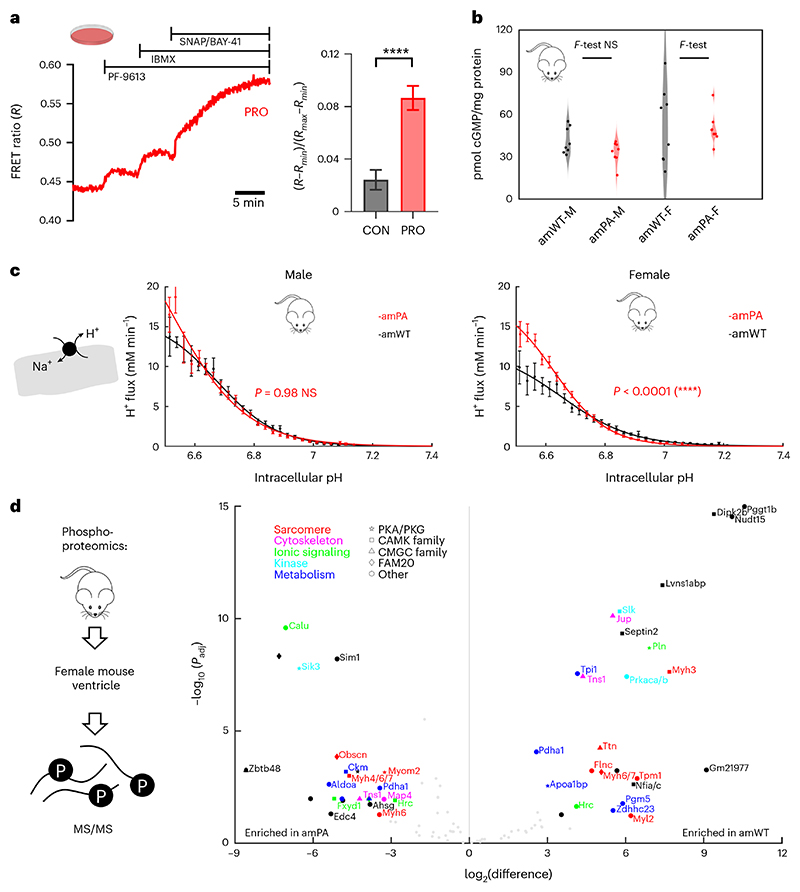
Disrupted propionyl-CoA handling evokes changes in protein phosphorylation. **a**, Representative cGMP FRET trace in NRVMs treated for 48 h with 3 mM propionate (PRO) to induce *Pde9a* expression, showing the effect of PDE9A-specific inhibitor (PF-9613; 100 μM), IBMX (100 μM) and SNAP/BAY-41 (50 μM and 5 μM, respectively). Double-normalised FRET signal in response to PF-9613 treatment at steady state, showing significant increase in propionate-treated (PRO) myocytes. Bar chart shows quantification of the effect of PF-9613 relative to untreated (CON) NRVMs. Hierarchical analysis of 88–117 myocytes from *n* = 4 isolations. Unpaired two-tailed *t*-test. ****P < 0.0001. Mean ± s.e.m. **b**, cGMP assay in murine cardiac lysates, normalized to protein content (*n* = 8 biologically independent samples per sex and genotype). Violin plots show distribution of measurements as a proxy of the scope for cGMP signaling. *F*-test was performed to compare variance in WT versus PA hearts (*P < 0.05). **c**, Transmembrane H^+^ fluxes generated by Na^+^/H^+^ exchange activity in myocytes isolated from mouse hearts. Female PA myocytes have higher NHE1 activity, which is consistent with a higher engagement of cGMP signaling triggered by natriuretic peptide signaling. Two-way ANOVA analysis of data from 20–35 myocytes from six isolations per category. Mean ± s.e.m. **d**, Phosphoproteomics of lysates prepared from female PA and WT hearts (*n* = 6 biologically independent samples per sex and genotype). Differentially abundant peptides (*P*_adj_ < 0.05), color-coded by type of protein. Shape of symbol indicates the most likely kinase (phosphosite functional score >3) for the given peptide substrate. Two-sample *t*-test, corrected for multiple comparisons. F, female; M, male; NS, not significant.

The most prominent propionate-related transcriptional response in amPA mice was a three-fold induction of *Pde9a* in females, the only major PDE gene affected in amPA hearts (Extended Data Fig. 8b). This is relevant to contractile function because previous studies had implicated PDE9A in diastolic dysfunction through its role in degrading cGMP triggered by natriuretic signaling^41,42^. Furthermore, the signaling consequences of *Pde9a* induction may synergize with raised diastolic Ca^2+^ in reducing SV in female PA hearts. *Pde9a* induction occurred without changes in transcript levels of natriuretic peptide genes, indicating that the net effect on cGMP signaling in female PA hearts is likely to downward (Extended Data Fig. 8c). To confirm that propionate-evoked *Pde9a* upregulation increases cGMP-degradative capacity, we cultured NRVMs with 3 mM propionate for 48 h, and the catalytic activity of PDE9A was measured using an intracellular cGMP-sensitive Förster resonance energy transfer (FRET) probe (cGI-500) from the FRET response to a selective PDE9A inhibitor PF-9613 (Fig. 6a). The PF-9613-sensitive component was three-fold higher in propionate-treated myocytes, confirming elevated PDE9A activity. To gain insight into the scope for cGMP signaling in amPA hearts, we assayed cGMP levels in cardiac lysates, which generates a snapshot of what is otherwise a dynamic signal (Fig. 6b). The widest range in cGMP was noted in WT female hearts, which could be explained by lower PDE9A activity allowing greater cGMP variability. A PA-related reduction in cGMP variability was detected in females only. Strikingly, this result was consistent with the sex-dependent effect of PA on *Pde9a* transcript levels (Fig. 5l).

In the heart, cGMP and cAMP signaling is intertwined^43^. A bellwether of cyclic nucleotide signaling is the activity of Na^+^/H^+^ exchange (NHE1). Since *Slc9a1*, the gene coding for NHE1, was not differentially expressed in PA mice, any change in transport activity is likely post-translational^44^. NHE1 activity^44^ was significantly faster in amPA myocytes from females but not males (Fig. 6c). Critically, the pH dependence of NHE1 markedly changed shape, as expected from post-translational modifications^45^. This finding is consistent with a previous study that showed NHE1 activation after ablating atrial natriuretic peptide signaling^46^. Because PDE9a is thought to selectively reduce cGMP from a natriuretic peptide trigger^41^, *Pde9a* induction may explain accelerated NHE1 in female PA hearts.

Next, we undertook an unbiased phosphoproteomic approach on female hearts to seek evidence for changes in phosphopeptides that may be substrates for PKG and other kinases. Twenty-seven serine/threonine (S/T) sites and three tyrosine (Y) sites (26 proteins) were more phosphorylated in amWT, and 26 other S/T sites and one Y site (21 proteins) were more phosphorylated in amPA (Fig. 6d and Supplementary Tables 6 and 7). Affected proteins included sarcomeric, cytoskeletal, kinases and those involved in ionic signaling and metabolic processes. Several sites were identified as potential substrates of PKA/PKG; noteworthy is phospholamban (Pln) enriched in WT. In amPA, the reduction in phospholamban phosphorylation at S16 is predicted to reduce SERCA activity, which may explain why raised diastolic Ca^2+^ does not produce a rise in CaT amplitude in female amPA myocytes. Additionally, several CAMK-family kinase substrates were affected, which may relate to the raised diastolic Ca^2+^ in female amPA (Fig. 4e). Net dephosphorylation at Myl2, Tpm1, Tns1, Ttn, Myh3 and Myh6 and net phosphorylation at Myom2 and two additional sites at Myh6 may affect myocardial stiffness. Changes to Pdha1, Tpi1, Pgm5, Apoa1bp, Aldoa and Ckm phosphorylation may be related to metabolic changes in PA^47^. The lower abundance of Pdha1 and Pln phosphopeptides in PA mice is consistent with a study of PDE9A inhibition in mice^41^. Taken together, our data indicate important changes in protein phosphorylation, including sites that can link *Pde9a* induction with contractile dysfunction.

### Histone acylation evoked by propionate/propionyl-CoA

**Extended Data Fig. 9:**
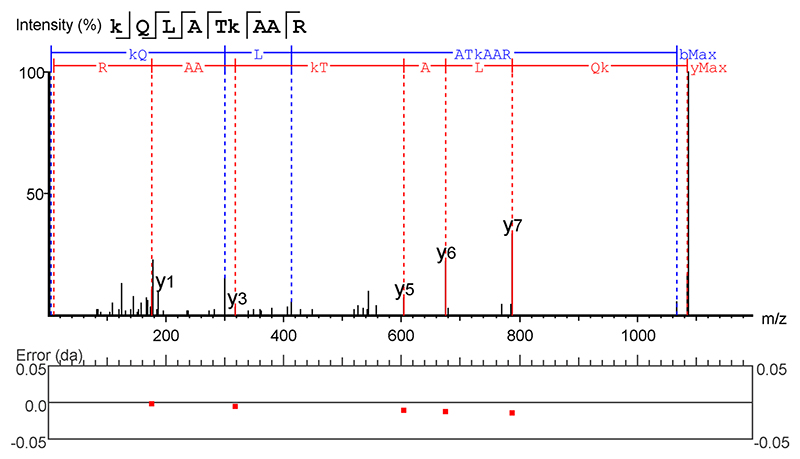
Exemplar histone LC-MS/MS spectrum. Exemplar MS spectrum of peptide 19–27 (from a sample treated under the condition ‘elevated propionate signalling’) showing ^13^C_1_-propionylation at K23.

**Fig. 7:**
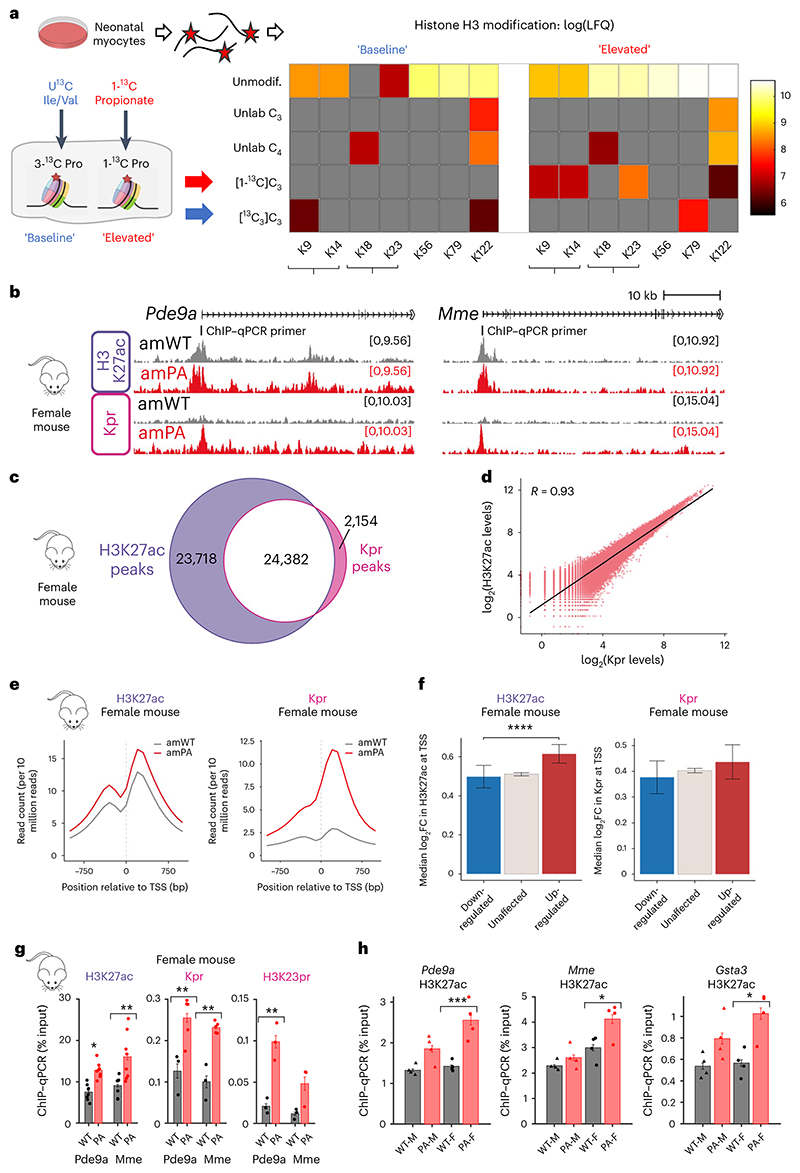
ChIP reveals increased histone acetylation and propionylation at propionate-responsive genes. **a**, ^13^C tracing of propionyl-CoA sourced from propiogenic substrates (isoleucine/valine) or exogenous propionate to identify propionylation sites on histone H3 under baseline and elevated propionate signaling, respectively. Lysine propionylation was identified by LC–MS/MS. Heat map shows label-free quantification intensity for the various lysines (*x* axis). The modifications include stable (unlabeled) propionylation (Pro) (‘unlab C_3_’) or (iso)butyration (‘unlab C_4_’) or propionylation with one labeled carbon from 1-^13^C-propionate (1-^13^C Pro) or three labeled carbons from ^13^C Ile/Val (3-^13^C Pro). Gray indicates no signal detected. Intensity is expressed as log_10_. Residues grouped by brackets are present on the same peptide fragment. **b**, Reference-normalised ChIP-seq in female amWT and amPA mouse hearts, using antibodies against histone H3K27ac and pan-propionylation (Kpr). ChIP-seq tracks are shown at the *Pde9a* and *Mme* promoter loci. **c**, Overlap of H3K27ac and Kpr peaks in female amWT mice. **d**, Correlation of H3K27ac and Kpr levels at H3K27ac peaks in female amPA mice. **e**, Metaplots showing the mean level of H3K27ac or Kpr at active gene promoters in female amWT or amPA mice relative to the transcriptional start site (TSS). **f**, Median log_2_(fold change (FC)) (amPA to amWT) in H3K27ac or Kpr levels for genes that are upregulated, downregulated or unaffected in PA. Error bars show 95% confidence intervals. ****P < 0.0001. **g**, ChIP–qPCR at the *Pde9a* and *Mme* promoters using antibodies against H3K27ac (*n* = 8 biologically independent samples per genotype), pan-Kpr (*n* = 3 amWT and *n* = 5 amPA biologically independent samples) and H3K23pr (*n* = 3 biologically independent samples per genotype). Locations of primer pairs are shown in **b**. Unpaired two-tailed *t*-tests. */** *P* < 0.05/<0.01. Mean ± s.e.m. **h**, ChIP–qPCR at the promoters of *Pde9a, Mme* and *Gsta3* for H3K27ac in male or female amWT and amPA mice. PA was associated with a significant increase in H3K27ac only in females. *n* = 4 biologically independent samples. Ordinary two-way ANOVA with Tukey’s multiple comparisons test. */*** *P* < 0.05/<0.001. Mean ± s.e.m. F, female; Ile, isoleucine; M, male; Unlab, unlabeled; Unmodif., unmodified; Val, valine.

We reasoned that the transcriptional responses in the hearts of PA mice relate to increased histone acetylation and propionylation. Whereas the role of histone acetylation in the heart is well established, less is known about histone propionylation in cardiac cells. To test this, we performed in vitro ^13^C tracing experiments in NRVMs using labeled valine and isoleucine (48-h treatment) or 1-^13^C-propionate (24-h treatment), followed by proteomic analyses of acid-extracted histones (Fig. 7a and Supplementary Table 8; exemplar MS spectra in Extended Data Fig. 9). Propionylation of histone H3 was detected at multiple lysine residues from the incorporation of ^13^C-propionate. This indicates that propionyl-CoA, derived from either ^13^C-BCAAs or ^13^C-propionate, can be used as a substrate for propionylation. A stable putative (iso)butyrylation at H3K18 and K122 was also detected. Intriguingly, histone propionylation displayed three distinct dynamic properties. First, unlabeled propionate was detected at H3K122, indicating a relatively stable modification, which only weakly incorporates label, even after 48-h treatment with heavy propionate. Second, H3K9 propionylation was exclusively labeled with ^13^C from BCAAs or propionate, suggesting high turnover within the treatment window. Finally, elevated propionate signaling (but not baseline) resulted in ^13^C-labeled propionylation of H3K14 and H3K23, indicating that increased modification of these residues may play a role in the nuclear response to exogenous propionate.

**Extended Data Fig. 10:**
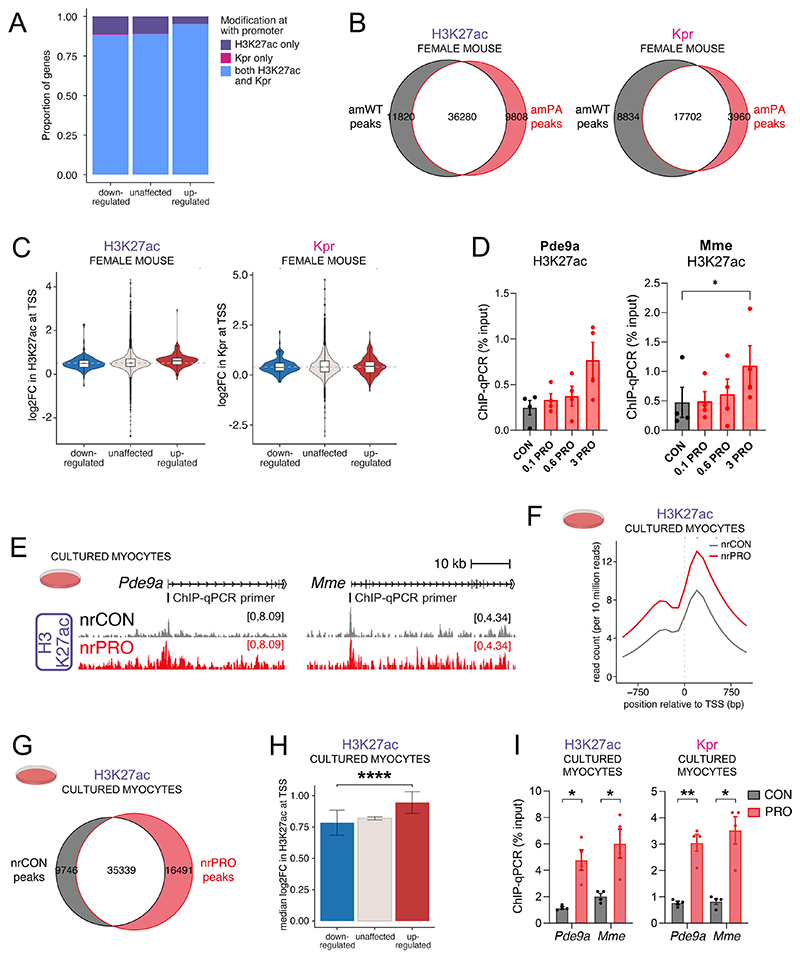
Chromatin immunoprecipitation reveals increased histone acetylation and propionylation at propionate-responsive genes. **(a)** Proportion of genes marked by a peak of H3K27ac, Kpr, or both modifications at the promoter, for unaffected and differentially expressed genes in amPA mice. **(b)** Overlap of H3K27ac or Kpr peaks in female amWT and amPA mice. **(c)** Log_2_-fold change in H3K27ac or Kpr levels in amPA mice relative to amWT mice at the promoters of active genes that are unaffected or sensitive to both PA *in vivo* and propionate *in vitro*. Dashed line shows the median log_2_-fold change for unaffected genes. Upper and lower hinges showing 25th and 75th percentile, respectively. Upper and lower whiskers extend to the largest and smallest datapoints within 1.5 times the interquartile range of either hinge. **(d)** Concentration-dependence of propionate effect on H3K27ac ChIP-qPCR at *Pde9a* and *Mme* promoters in NRVMs. N = 4 matched biologically independent samples per condition from 4 isolations. Repeated measures one-way ANOVA with Dunnett’s multiple comparisons test. * P < 0.05. Mean ± SEM. **(e)** Referencen-ormalised ChIP-seq for H3K27ac in cultured neonatal rat ventricular myocytes, either untreated (nrCON) or treated with 6 mM propionate (nrPRO) for 48-h. ChIP-seq tracks are shown at *Pde9a* and *Mme*. **(f)** Metaplots showing the mean level of H3K27ac at active gene promoters in cultured neonatal rat ventricular myocytes treated with 6 mM propionate (nrPRO) for 48-h or control (nrCON). **(g)** Overlap of H3K27ac peaks in untreated (nrCON) NRVMs or myocytes treated with 6 mM propionate for 48-h (nrPRO). **(h)** Median log_2_-fold change in H3K27ac in treated (nrPRO) versus untreated (nrCON) myocytes for genes that are up-regulated, down-regulated or unaffected by propionate treatment *in vitro*. Error bars show 95% confidence intervals. **** P < 0.0001. **(i)** ChIP-qPCR at the *Pde9a* and *Mme* promoters for H3K27ac and Kpr cultured myocytes, showing increase in nrPRO, relative to nrCON. N = 4 biologically independent samples per condition from 4 isolations. */** P < 0.05/0.01. Mean ± SEM.

To test for epigenetic changes triggered by histone acylation, we performed reference-normalised chromatin immunoprecipitation-sequencing (ChIP-seq) on formaldehyde-fixed ventricular tissue from female amPA and amWT mice, using antibodies recognising H3K27ac and pan-propionyllysine (Kpr) (Fig. 7b). A pan-Kpr antibody was preferred over residue-specific alternatives because it captures the effect of PA on multiple residues. Histone acetylation and propionylation co-localised at the promoters of many active genes, including *Pde9a* and *Mme* (Fig. 7c), and correlated strongly (Fig. 7d), which would be consistent with both moieties being deposited at the same genomic loci by the same lysine acyltransferases^41^. Moreover, genes that were upregulated in PA mice were more likely to be marked with both H3K27ac and propionylation (Extended Data Fig. 10a). However, the overlap was not complete, with some genes showing a peak of H3K27ac but not propionylation.

Globally elevated histone acetylation and propionylation were observed in the hearts of PA mice (Fig. 7e), arguing that endogenously sourced propionate can induce direct propionylation and net acetylation. Although the level of modifications was increased, the number of peaks observed was broadly similar (Extended Data Fig. 10b), indicating that elevated propionate does not result in the targeting of acylation to novel genomic loci. To determine whether increased histone acylation could explain gene expression changes in PA mice, we studied changes in H3K27ac and Kpr levels at the promoters of DEGs. Indeed, promoters showed an increase in both modifications, irrespective of their transcriptional response. Strikingly, the increase in H3K27ac was significantly greater at upregulated DEGs, indicating a correlation between the extent of histone acetylation and transcriptional response (Fig. 7f and Extended Data Fig. 10c; note: there was not enough statistical power in the analysis of DEGs with changed Kpr levels at their promoters to perform the analogous analysis). ChIP–qPCR for the upregulated genes *Pde9a* and *Mme* confirmed significantly higher levels of H3K27ac and Kpr in female amPA mice (Fig. 7g). We corroborated this using a site-specific propionylation antibody recognising H3K23pr (Fig. 7g), a major propionylation site in cardiomyocytes. A second batch of ChIP–qPCR experiments compared histone modifications between male and female mice to seek a sex dependence. In contrast to the significant PA-associated increase in H3K27ac in females, the level of H3K27ac at promoters of *Pde9a, Mme* and *Gsta3* was unaffected in male PA mice (Fig. 7h). This finding suggests that the stronger transcriptional response in female PA mice relates to more profound changes in histones and underlying metabolic changes.

Finally, we tested whether the histone modifications in female PA mice could be evoked in vitro. In propionate-treated NRVMs, we found that histone acetylation at *Pde9a* and *Mme* promoters was propionate dependent (Extended Data Fig. 10d) and consistent with the effect of propionate on H3K9ac (Fig. 5h; K_i_ ~ 0.6 mM). ChIP-seq in NRVMs treated with 6 mM propionate for 24 h (that is, attaining saturating levels of propionyl-CoA) revealed an increase in H3K27ac at active gene promoters, indicating that the chromatin changes observed in female amPA hearts could be attributed to elevated propionate (Extended Data Fig. 10e–g). As with mice, in vitro propionate treatment evoked a stronger change in H3K27ac among promoters of upregulated genes, providing a basis for differential gene expression (Extended Data Fig. 10h). ChIP–qPCR experiments confirmed that upregulated genes *Pde9a* and *Mme* showed a strong increase in H3K27ac and Kpr levels (Extended Data Fig. 10i).

## Discussion

This study established a link between disrupted propionate metabolism and epigenetic changes in the heart. The transmission of the sex-dependence of PA consequences on the metabolic disturbances, histone modifications, transcriptional responses and cardiac derangements in PA mice suggests an epigenetic basis for the cardiac actions of propionate. In patients with PA, multiple organs are affected, but deaths are commonly cardiac^19^. An indication that perturbed propionate can disrupt cardiac function came from comparing PA with methylmalonic acidaemia (MMA), which affects the enzyme downstream of PCC and has a less definitive cardiac disease phenotype^19^. Here we provide the first demonstration, to our knowledge, that an endogenous source of propionate can modify histones in the heart, and we show how these modifications trigger transcriptional responses relevant to cardiac function. Our results describe a novel signaling axis, relevant to metabolic diseases of the heart whenever a rise in propionyl-CoA is implicated. Given that propiogenic substrates and gut bacteria can yield substantial quantities of propionate, these responses may have broad impact beyond PA.

Our study noted a greater excess of propionyl-CoA and the acyl-CoA intermediates of propiogenic substrate catabolism in female PA hearts, compared to male counterparts, despite attaining similar plasma levels of propionate and propionyl-carnitine (a surrogate of systemic propionate-load). Sexual dimorphism in CoA handling was noted previously^48^ and likely relates to sexual maturity; if so, it will not normally manifest in patients with PA who are typically pediatric. Nonetheless, the mechanistic causes of the sex-dependence may indicate directions for better disease management. The metabolic disturbance in PA mice was relatively limited (~130 metabolites) and included 22 metabolites showing a sex dependence. The most prominent sex-dependent response was an increase in β-alanine and its dipeptides (carnosine and anserine) in male PA hearts. β-alanine, a downstream derivative of propionyl-CoA, is a rate-limiting precursor of carnosine^35^. Through the actions of testosterone, male mice accumulate more carnosine in skeletal muscle^49^, and a similar mechanism is thought to operate in the heart^28^. We traced propionate to carnosine in vitro and propose that this pathway is scaled-up in male PA hearts. Raised carnosine protects from oxidative stress^28^, improves mitochondrial function and facilitates the clearance of propionate-related metabolites. Indeed, loading propionate-stressed myocytes in vitro with β-alanine reduced the 2-methylcitrate/citrate ratio (a PA marker) as well as propionyl-CoA, isovaleryl-CoA, (iso)butyryl-CoA and tiglyl-CoA.

We observed that PA hearts had increased histone propionylation and acetylation, typically deposited at common genomic loci. Isotope tracing demonstrated that exogenous propionate and propiogenic substrates (BCAAs) convert to propionyl-CoA, which then modifies histone H3 at various lysine residues. Under baseline metabolic conditions, propionyl-CoA propionylates H3K9 and H3K122, whereas, under circumstances of elevated propionyl-CoA, H3K23 and H3K14 are targeted. The increase in histone acetylation could be explained in terms of the inhibitory effect of propionate on HDACs^14,17,18^ or an activatory effect of propionyl-CoA on p300 acetylase^13^. In support of net histone acetylation, many of the transcriptional responses to propionate in vitro were also evoked by treatment with butyrate, which raises acetylation but not propionylation. Propionate anions inhibit HDACs with an inhibitory constant in the range of hundreds of micromolar^13^, and, if this was a relevant mechanism, the intracellular concentration of propionate in vivo would have to be substantially higher than in plasma. This scenario would be expected if propionate was produced by cardiac metabolism. Moreover, it is possible that propionyl-CoA/propionate levels are highly compartmentalised in cardiac nuclei^16^.

Consistent with the sex-dependent metabolic disturbance, we described more profound histone modifications and stronger transcriptional responses in female PA hearts. Genes induced in female PA hearts include *Pde9a* and *Mme*, part of natriuretic peptide-triggered cGMP signaling pathway^50,51^. At least some of the changes in the cardiac phosphoproteome may be linked to a downscaling of cGMP signals arising from *Pde9a* induction. Among differentially abundant phosphopeptides were contractile elements, whose phosphorylation status may affect myocardial stiffness. It was noteworthy that female PA mice had a more pronounced contractile dysfunction, characterised by a raised ESV and reduced SV. We excluded systolic dysfunction because the amplitude of Ca^2+^ transients and cell-shortening were unaltered in PA. However, raised diastolic Ca^2+^ in female PA myocytes would lead to less complete myofilament deactivation during diastole. The observation that elevated diastolic Ca^2+^ did not produce the expected rise in CaT amplitude indicates that the SR takes up Ca^2+^ less avidly in PA, which likely relates to the right-shift in the Ca^2+^activation curve of SERCA. A plausible explanation relates to changes in phospholamban phosphorylation, but further work is required to dissect this pathway. Overall, the remodeling in female PA hearts has features of diastolic dysfunction. Supporting this model, previous studies linked *Pde9a* induction to diastolic dysfunction^41^ and low PKG activity to raised resting tension and impaired relaxation^42^. Notwithstanding the contractile phenotype in female PA mice, it is important to note that some important clinical signatures of diastolic dysfunction were not observed; for example, there was no increase in E/E′ ratio or evidence for pulmonary edema. It is possible that a more severe phenotype will develop with larger and longer propionyl-CoA disturbances.

Because PA is defined as an acidosis, it is intuitive to speculate about a role for reduced pH_i_ in the contractile dysfunction. Moreover, we observed an increase in NHE1 activity in female PA myocytes. However, measurements of resting pH_i_ revealed no effect of PA, arguing against a role of disturbed intracellular acid–base balance in PA cardiac dysfunction. Whereas NHE1 is the major pH regulator responsible for extruding large acid loads from the cytoplasm, transporters of the Na^+^-HCO_3_^−^ family are critical in keeping resting pH_i_ near physiological levels. Nonetheless, higher NHE1 activity in female PA mice may increase susceptibility to Na^+^ overload during acid stress, which would be proarrhythmogenic. Further experiments in stressed hearts would provide insight into this additional disease mechanism.

Although ChIP analyses revealed a wide scope for propionate to modify histones, the coupling between histone marks and transcriptional activity is highly non-stoichiometric. An increase in histone acylation may produce a noticeable transcriptional change at only a small subset of genes. Indeed, we measured a robust response at the transcript level for a subset of genes only. Although we observed an increase in H3K23pr at the promoter of *Pde9a* and *Mme* in female PA mice, further work is needed to link specific histone residues to transcriptional responses, and to determine whether their acetylation and propionylation cause moiety-specific responses. To that end, the generation of site-specific antibodies is essential.

Although *Pde9a* induction is a formidable candidate for cardiac dysfunction, there will be a myriad of coinciding transcriptional responses in PA, each making a contribution to the disease state. We speculate that a targeted approach of silencing any isolated transcriptional response may not necessarily reverse all PA-related changes. The therapeutic priority should, instead, focus on reducing the total propionyl-CoA load^52^. We postulate that raising β-alanine could be therapeutic, possibly as a diet enriched in the free amino acid or its dipeptides.

We conclude that disruptions to metabolism that raise propionyl-CoA can have profound effects through epigenetic actions that impact contraction in a range of cardiometabolic diseases. Metabolic stress, such as that emerging in diabetes or with abnormal gut microbiome activity, may amplify propionate-generating pathways and increase histone acylation in the heart. It will, therefore, be important to investigate these histone responses in the broader context of cardiac disease.

## Methods

### Experimental models and sample collection

All protocols were conducted in accordance with the University of Oxford Animal Welfare and Ethical Review Body and authorized by the UK Animals (Scientific Procedures) Act of 1986. Animals were killed using an appropriate method of humane killing (Schedule 1) or anesthetic overdose. Experimental protocols and data analysis were randomised, but blinding was not always possible owing to most work being conducted by a single operator.

#### In vitro cardiomyocyte models

Supporting in vitro experiments were performed on neonatal or adult rat ventricular myocytes (ARVMs) treated with exogenous propionate. For this purpose, rat hearts were used as a refinement measure because they yield a greater number of cells for culture than mouse hearts, yet both species have similar cardiac physiology and gene regulation^47^. Experiments were performed on ventricular myocytes isolated from either 275–300-g adult or postnatal day 1–2 (P1/P2) neonatal Sprague–Dawley (SD) rats (Charles River Laboratories). The procedures for isolating ventricular myocytes are provided below.

#### In vivo model of disrupted propionate metabolism

Most experiments were performed on adult male and female mice that were 8 weeks of age, from the FVB *Pcca*^−/−^ A138T line generated by Guenzel et al.^23^. A small selection of experiments was performed in 20-week-old mice and indicated accordingly. Mice were bred and housed in individually ventilated cages, and all animals had ad libitum access to water and food (Teklad, 2918, global 18% protein rodent diet). Mice used were either WT (*Pcca*^+/+^ A138T) for control experiments or homozygous (*Pcca*^−/−^ A138T) animals, referred to as amWT and amPA, respectively. Only heterozygous or WT dams were used for breeding, and genotyping by PCR was performed as previously described^23^.

#### Tissue harvesting and sample collection

Most experiments were conducted on ventricular myocytes freshly isolated in Langendorff mode or on cryoground tissues that were snap frozen by liquid nitrogen, with the exception of ChIP, which was performed on formaldehyde-crosslinked hearts fixed in Langendorff mode. To harvest tissues, mice were killed humanely by intraperitoneal injection of an appropriate volume adjusted for body weight of sodium pentobarbitone (stock 200 mg ml^−1^) pre-mixed at a 2:1 ratio with sodium heparin (stock 5,000 IU ml^−1^). Upon confirmation of anesthesia, the beating heart was rapidly excised by incising the aorta, and blood was allowed to pool in the thoracic cavity. The heart was promptly arrested in ice-cold 1× PBS; the atria was dissected away; and the total ventricle was blotted dry before being snap frozen in liquid nitrogen. Pooled whole blood in the thoracic cavity was immediately collected (within 30 s of excising the heart) by gentle aspiration with a 1-ml syringe and injection into lithium heparin tubes (BD Vacutainer, 368495). Blood was kept on ice and promptly centrifuged at 1,200 relative centrifugal force (RCF) at 4 °C for 10 min to collect plasma, and the red cells and buffy coat were discarded. All samples were stored at −80 °C until use. Further details are provided in subsequent sections.

#### Human plasma samples

Patient parent/guardian written consent for permitting research studies was obtained and recorded in the Studies of Inherited Metabolic Diseases database. Measurements and analyses were performed according to guidelines set by the Royal College of Pathologists and the Great Ormond Street Hospital (GOSH) NHS Foundation Trust. As part of ongoing clinical care, plasma samples are collected from patients with PA for measurements of propionate and derivatives. Once these tests are completed, surplus acellular plasma is anonymized and used for diagnostic measurements, in line with GOSH departmental policy and approvals. Samples are non-identifiable, and no additional information was obtained beyond the measurements presented herein.

### Isolation of adult mouse ventricular myocytes

Isolation of primary ventricular myocytes was as previously described^39^ through Langendorff-perfused hearts using a combination of enzymatic (~1 mg ml^−1^ collagenase, Worthington Biochemical; 0.025 mg ml^−1^ type XIV protease, P5147, Sigma-Aldrich/Merck) and mechanical dispersion. The isolation solution for adult mice contained (in mmol L^−1^): 130 NaCl, 5.6 KCl, 5 HEPES, 0.45 NaH_2_PO_4_, 10 glucose, 20 taurine and 3.5 MgCl_2_; pH adjusted to 7.40 at 37 °C with NaOH. The isolation solution for adult rats contained (in mmol L^−1^) (unless stated otherwise): 120 NaCl, 4 KCl, 10 HEPES, 2 NaH_2_PO_4_, 11 glucose, 20 taurine, 1.2 MgCl_2_ and 0.0173% v/v pyruvic acid; pH adjusted to 7.40 at 37 °C with NaOH. After digestion of the heart, ventricular tissue was minced in isolation solution containing 200 μM CaCl_2_ and 1% BSA (w/v) to quench enzymatic activity. Cells were filtered through a 400-μm nylon mesh, and the remaining tissue was further dissociated through subsequent digestions for 5 min per round at 37 °C combined with gentle trituration. Cells were pelleted by centrifugation at 40 RCF for 1 min, washed once in isolation solution containing 500 μM CaCl_2_ and then finally in 1 mM CaCl_2_ before use. Myocytes were allowed to rest for at least 30 min before use, and only quiescent rod-shaped myocytes were selected for experiments.

### Isolation of NRVMs

NRVMs were isolated from postnatal day 1 and 2 (P1/P2) SD rats as previously described^39^. Hearts were pooled from a litter of 12 pups for the isolation of myocytes. After Schedule 1, hearts were excised and washed in ice-cold 1× ADS buffer containing (in mmol L^−1^): 106 NaCl, 5.3 KCl, 20 HEPES, 0.8 NaH_2_PO_4_, 0.4 MgSO_4_ and 5 glucose; pH adjusted to 7.40 at 37 °C with NaOH. After removing the atria under a stereoscopic microscope, the ventricles were finely minced and digested with 0.45 mg ml^−1^ type A collagenase (Roche) and 1.25 mg ml^−1^ pancreatin (Sigma-Aldrich/Merck) for 5 min at 37 °C with gentle stirring. The supernatant from the first digestion was discarded. The remaining tissue was digested for 20 min at 37 °C with gentle stirring. The supernatant, containing myocytes and fibroblasts, was collected, and enzymatic activity was quenched using newborn calf serum (NCS). The cells were then centrifuged at 250 RCF for 5 min in a swing-out rotor centrifuge, and the pellet was resuspended in fresh pre-equilibrated medium (see below) containing 10% horse serum (HS) and 5% NCS and stored in a humidified 5% CO_2_ incubator. The digestion/collection process was repeated until all ventricular tissue was digested. The final supernatant was ‘preplated’ on untreated 10-cm Petri dishes for 2 h at 37 °C to promote the attachment of fibroblasts, after which the myocyte-enriched supernatant was collected and seeded on the appropriate cell culture platform pre-coated with fibronectin. Cells were maintained in a humidified 5% CO_2_ incubator at 37 °C and cultivated in media as described below.

### Media for standard cell culture

Stock media was based on 20% (v/v) M199 (Sigma-Aldrich/Merck, M4530) and 80% (v/v) NaHCO_3_-free DMEM (Sigma-Aldrich/Merck, D7777) supplemented with 24 mM NaHCO_3_ and 20 mM NaCl (to adjust osmolality to physiological levels). Then, 1% (v/v) penicillin–streptomycin was included in media (100 U ml^−1^ penicillin and 100 μg ml^−1^ streptomycin; Sigma-Aldrich/Merck, P4333). All media was prepared using ultra-pure water, sterile filtered (0.22 μm) and stored at 4 °C. Before use, media was pre-equilibrated in a humidified 5% CO_2_ incubator for a minimum period of 1 h to achieve equilibration of pH and temperature^53^. NRVMs were isolated in stock media containing 10% HS and 5% NCS. On day 1 after isolation, serum was reduced 10-fold (1% HS and 0.5% NCS), and, on day 2, cells were serum starved. NRVMs were treated on day 3 for 24–48 h under serum-free conditions in treatment media prepared using NaHCO_3_-free DMEM (D7777) supplemented with 24 mM NaHCO_3_ and, where appropriate, sodium propionate or sodium butyrate, with various concentrations of NaCl to balance osmolality between treatment media. Dulbecco’s PBS containing calcium and magnesium (Gibco, 14040-133) was used for washing cells between media changes.

### Custom media for ^13^C tracing

All media was prepared using ultra-pure water, sterile filtered (0.22 μm) and stored at 4 °C. Custom media was prepared from commercially available DMEM powder that does not contain glucose, amino acids, sodium bicarbonate, sodium pyruvate and Phenol Red (US Biologicals, D9800-20A). The final concentration of amino acids was based on classical DMEM formulations. ‘Stock tracer media’ was prepared from DMEM powder and supplemented with (in mmol L^−1^): 21.1 glucose, 0.399 L-arginine•HCl, 0.2 L-cysteine•2HCl, 0.399 glycine, 0.219 L-histidine•HCl•H_2_O, 0.8 L-isoleucine, 0.8 L-leucine, 0.8 L-lysine•H_2_O, 0.201 L-methionine, 0.4 L-phenylalanine, 0.4 L-serine, 0.798 L-threonine, 0.078 L-tryptophan, 0.397 L-tyrosine•2Na•2H_2_O, 0.802 L-valine, 24.4 NaHCO_3_, 0.042 Phenol Red•Na, 1 sodium pyruvate and 19.9 NaCl. Then, 1% (v/v) penicillin–streptomycin (final concentration 100 U ml^−1^ penicillin and 100 μg ml^−1^ streptomycin; Sigma-Aldrich/Merck, P4333) and 1% (v/v) L-glutamine (final concentration 2 mM L-glutamine; GlutaMAX, Thermo Fisher Scientific, 35050-038) was added to all freshly prepared media, in serum-free conditions. This custom media was used to prepare different tracer media containing either alone or in combination, where indicated, 0.8 mM U^13^C_6_ L-isoleucine (Cambridge Isotope Laboratories, CLM-2248-H), 0.8 mM U^13^C_5_ L-valine (Sigma-Aldrich, 758159) and/or 1 mM 1-^13^C-sodium propionate (Cambridge Isotope Laboratories, CLM-771-PK). Where appropriate, custom media was prepared without L-histidine and supplemented with 5 mM carnosine.

### Plasma propionate and plasma acylcarnitine mass spectrometry measurements

Analysis of plasma propionate and plasma acylcarnitine derivatives was performed by tandem mass spectrometry at GOSH, London.

#### Plasma propionate

Plasma specimens were prepared by adding to 50 μl of plasma 10 μl of saturated analar sodium bicarbonate and 10 μl of 0.5 mM sodium propionate 1-^13^C as the heavy isotope internal standard. After protein precipitation with 400 μl of absolute ethanol, the separated liquid extract was blow-dried with nitrogen gas at 38 °C and reconstituted in 200 μl of 1:1 methanol:water mixture. Then, 20 μl of reconstituted extract, equivalent to 4 μl of plasma, was injected into the mobile phase stream of 50% methanol in water with 0.02% NH_3_. The mobile flow rate was 5 μl min^−1^ over a period of 2.0 min, rising to 300 μl min^−1^ by 2.2 min and maintained at a flow rate of 300 μl min^−1^ until 3.0 min. For the analysis by tandem mass spectrometry, electrospray negative ionization was employed in the multiple reaction monitoring mode with the following mass transitions to identify and quantify propionate: CH_3_CH_2_COO^−^ *m*/*z* 73 > 73 and CH_3_CH_2_^13^COO^−^ *m*/*z* 74 > 74.

#### Plasma acylcarnitines

The acylcarnitine method employed was based on a published method^54^. Acylcarnitines were analyzed on 5 μl of plasma as their butyl-ester derivatives. A combined mixture of heavy isotope internal standards was added to the plasma specimens before butylation with HCl butanol. The final concentration of the free carnitine internal standard was 100 μmol, and the propionylcarnitine internal standard was 5 μmol in the plasma specimen. For this study, the free carnitine and propionylcarnitine were analyzed in positive ion mode using precursor-ion (parent-ion) scan. As all acylcarnitine species have a common product (*m*/*z* 85), the precursor-ion mode allows analysis of a wide complete range of different butylated acylcarnitine derivatives. The butylated precursor-free carnitine ion (*m*/*z* 218) was quantified against a 9-deuterium-labeled heavy isotope-free carnitine with a butylated precursor ion (*m*/*z* 227). The butylated precursor propionate ion (*m*/*z* 274) was quantified against a 3-labeled heavy isotope propionate butylated precursor ion (*m*/*z* 277). The processed liquid sample is introduced into the ion source using positive mode electrospray ionization at atmospheric pressure. The mobile phase was 80% methanol in water with a flow rate of 5 μl min^−1^ over a period of 2.0 min, rising to 300 μl min^−1^ by 2.2 min and maintained at a flow rate of 300 μl min^−1^ until 3.0 min.

### Unlabeled metabolomic analyses

Analyses were performed in the Department of Chemistry at the University of Oxford.

#### Tissue preparation

Tissue metabolites were extracted from cryoground heart or liver tissue by adding 500 μl of 80% (v/v) MS-grade MeOH to 50 mg of tissue. The tissue underwent 2–3 rounds of brief homogenization (~10 s) and then was centrifuged at 21,000 RCF for 20 min at 4 °C. Pelleted insoluble material was discarded, and the supernatant was processed with 10-kDa cutoff ultra-centrifugation filter columns (Amicon Ultra-0.5 Centrifugal Filter Units, Merck/Sigma-Aldrich, UFC501096) at 21,000 RCF for 20 min at 4 °C. The infranatant was transferred to Protein LoBind 1.5-m tubes (Eppendorf) and kept on ice. Nucleic acid concentration was estimated with a spectrophotometer (NanoDrop, Thermo Fisher Scientific), and all tissue samples were diluted to the sample with the lowest concentration using 80% MeOH, before being processed downstream.

#### Plasma preparation

Plasma metabolomics samples were prepared by adding 230 μl of MS-grade 1-butanol:methanol (1:4 v/v) to every 100 μl of plasma. The sample was vortexed well and processed with 10-kDa cutoff ultra-centrifugation filter columns at 21,000 RCF for 30 min at 4 °C. The infranatant was then processed downstream.

#### IC–MS

Ion-exchange chromatography^55^ was performed using an ICS-5000 HPLC system incorporating an electrolytic anion generator (KOH based) that was programmed to produce an OH^−^ gradient over 37 min. An inline electrolytic suppressor removed OH^−^ ions and cations from the post-column eluent stream before MS analysis (Thermo Fisher Scientific, Dionex AERS 500). A 10-μl partial loop injection was used for all analyses, and the chromatographic separation was performed using a Thermo Fisher Scientific Dionex IonPac AS11-HC 2 × 250 mm, 4-μm particle size column with a Dionex Ionpac AG11-HC 4-μm 2 × 50 guard column inline. The IC flow rate was 0.250 ml min^−1^. The total run time was 37 min, and the hydroxide ion gradient was comprised as follows: 0 min, 0 mM; 1 min, 0 mM; 15 min, 60 mM; 25 min, 100 mM; 30 min, 100 mM; 30.1 min, 0 mM; 37 min, 0 mM. Analysis was performed in negative ion mode using a scan range from *m/z* 60 to *m*/*z* 900 and resolution set to 70,000. The tune file source parameters were set as follows: sheath gas flow 60 ml min^−1^; aux gas flow 20 ml min^−1^; spray voltage 3.6 V; capillary temperature 320 °C; S-lens RF value 70; heater temperature 350 °C. The automatic gain control (AGC) target was set to 1 × 10^6^ ions, and the Max IT value was 250 ms. The column temperature was kept at 30 °C throughout the experiment. Full scan data were acquired in continuum mode. The derivatisation of samples was based on a modified version of the Waters AccQ-Tag method^56^. A Thermo Ultimate 3000 UHPLC system was coupled directly to a Q Exactive Hybrid Quadrupole-Orbitrap mass spectrometer. A 5-μl partial loop injection was used for all analyses with pre-injection and post-injection wash program. A Waters AccQ-Tag column (2.1 × 100 mm) was used with a flow rate of 0.5 ml min^−1^. The total run time was 9.5 min. Mobile phase A and B comprised commercially available AccQ-Tag reagents prepared as recommended by Waters. The gradient elution program was modified from the published AccQ-Tag method as follows: 0 min, 0.1% B; 0.54 min, 9.1% B; 5.74 min, 21.2% B; 7.74 min, 59.6% B; 8.04 min, 90% B; 8.05 min, 90% B; 8.64 min, 0% B; 9.5 min, 0.1% B. The column temperature was kept at 40 °C throughout the experiment. Mass spectrometry analysis was performed in positive ion mode separately using a scan range from *m*/*z* 70 to *m*/*z* 1,050 and resolution set to 70,000. The tune file source parameters were set as follows: sheath gas flow 60 ml min^−1^; aux gas flow 20 ml min^−1^; spray voltage 3.6 V; capillary temperature 320 °C; S-lens RF value 70; heater temperature 350 °C. Full MS settings were AGC target 3 × 10^6^ ions and the Max IT value 200 ms. Full scan data were acquired in continuum mode.

#### Data processing and statistics

Raw data files were processed using Progenesis QI (Waters) and included alignment of retention times, peak picking, characterising multiple adduct forms and metabolite identification using authentic standards. Matching of retention times (1-min window), accurate mass values (<5 ppm), relative isotope abundances (>90%) and fragmentation patterns were used for metabolite identifications. Where measured, fragmentation patterns were matched to at least the base peak and two additional peak matches in the MS/MS spectrum to within 12 ppm (the top 10 data-directed fragmentation method could not provide fragment ions for all ions in the MS 1 spectrum). MetaboAnalyst^57^ (https://www.metaboanalyst.ca/) was used for further data processing and statistical analysis of the data. *P* values were false discovery rate (FDR) corrected (Benjamini–Hochberg).

### ^13^C tracing metabolomic analyses

Analyses were performed in the Department of Chemistry at the University of Oxford.

### Sample preparation

Samples for ^13^C tracing metabolomics were performed in cultured NRVMs with custom media as described above (‘isolation of neonatal ventricular myocytes’ and ‘^13^C tracing custom media’). Between 2 million and 2.5 million NRVMs were seeded per 60-mm Petri dish for each condition. On day 3 after isolation, NRVMs were treated with culture media for 48 h. A total of 400 μl of ice-cold MS-grade methanol was used for each Petri dish to lyse cells. The lysate was scraped and collected into 1.5-ml tubes and centrifuged at 21,000 RCF for 30 min at 4 °C. The supernatant was collected and metabolites prepared after 10-kDa cutoff ultra-centrifugation as described above under section ‘Unlabeled metabolomic analyses’.

### Anion-exchange chromatography–mass spectrometry

Ion-exchange chromatography was performed using an ICS-5000 HPLC system coupled to a high-resolution Q Exactive Orbitrap mass spectrometer (Walsby-Tickle et al.^55^). The IC-5000 system incorporated an electrolytic anion generator (KOH based) programmed to produce an OH^−^ gradient over 37 min. An inline electrolytic suppressor removed OH^−^ ions and cations from the post-column eluent stream before MS analysis (Thermo Fisher Scientific, Dionex AERS 500). A 10-μl partial loop injection was used for all analyses, and the chromatographic separation was performed using a Thermo Fisher Scientific Dionex IonPac AS11-HC 2 × 250 mm, 4-μm particle size column with a Dionex Ionpac AG11-HC 4-μm 2 × 50 guard column inline. The IC flow rate was 0.250 ml min^−1^. The total run time was 37 min, and the hydroxide ion gradient was comprised as follows: 0 min, 0 mM; 1 min, 0 mM; 15 min, 60 mM; 25 min, 100 mM; 30 min, 100 mM; 30.1 min, 0 mM; 37 min, 0 mM. Mass spectrometry analysis was performed in negative ion mode using a scan range from *m*/*z* 60 to *m*/*z* 900 and a resolution set to 70,000. The tune file source parameters were assessed: sheath gas flow 60 ml min^−1^; aux gas flow 20 ml min^−1^; spray voltage 3.6 kV; capillary temperature 320 °C; S-lens RF value 70; heater temperature 350 °C. AGC target was set to 1 × 10^6^ ions, and the Max IT value was 120 ms. The column temperature was kept at 30 °C throughout the experiment. Full scan data were acquired in continuum mode.

### Reversed-phase chromatography–mass spectrometry of derivatized samples

Samples were derivatied using a modified version of the Waters AccQ-Tag method (Salazar et al.^33^). Reversed-phase liquid chromatography–mass spectrometry (RPLC–MS) analysis of derivatized samples was also performed using the Acquity UPLC system liquid chromatograph (Waters) coupled directly to a Xevo G2-XS QTOF high-resolution tandem mass spectrometer. A 5-μl partial loop injection was used for all analyses with pre-injection and post-injection wash programs. A Waters AccQ-Tag column (2.1 × 100 mm) was used with a flow rate of 0.6 ml min^−1^. The total run time was 9.5 min. Mobile phases A and B comprised commercially available AccQ-Tag reagents prepared as recommended by Waters. The gradient elution program was modified from the published AccQ-Tag method as follows: 0 min, 0.1% B; 0.54 min, 9.1% B; 5.74 min, 21.2% B; 7.74 mins, 59.6% B; 8.04 min, 90% B; 8.05 min, 90% B; 8.64 min, 0% B; 9.5 min, 0.1% B. The column temperature was kept at 50 °C throughout the experiment. Mass spectrometry analysis was performed in positive ion mode using a scan range from *m*/*z* 150 to *m*/*z* 1,200. The centroid data were collected in MSE mode, and the time was set to 0.2 s, *t*. The tune file source parameters were set as follows: source temperature 140 °C; cone gas flow 50 L h^−1^; desolvation gas flow 800 L h^−1^. Cone voltage and capillary voltage were set to 40.0 V and 2.0 kV in negative ion mode, respectively. Full scan data were acquired in continuum mode.

### Data processing and analysis of the mass spectrometry data

Raw data files were converted into mzML format using ProteoWizard MSConvert^58^ before data processing and isotopologue extraction using El Maven^59^. Selected metabolites were identified using an in-house database of authentic metabolite standards by matching accurate mass values and chromatographic retention times from the experimental samples with database values. Peaks were aligned across samples in El Maven using standard settings, and a 5-ppm *m*/*z* window was used for extracted ion chromatograms for each isotopologue. Summed peak area abundances and individual isotopologue abundances for each identified metabolite were extracted and compared across each sample.

### Acyl-CoA and ^13^C tracing metabolomic analyses

Analyses were performed in the Babraham Institute.

### Sample preparation

Samples for acyl-CoA analysis were performed in cultured NRVMs with custom media as described above (‘isolation of neonatal ventricular myocytes’ and ‘^13^C tracing custom media’). Between 2 million and 2.5 million NRVMs were seeded per 60-mm Petri dish for each condition. On day 2 after isolation, NRVMs were serum starved and, on day 4, treated with custom media for 24 h. A total of 1 ml of ice-cold MS-grade 10% trichloroacetic acid (TCA) (v/v) was used for each Petri dish for cell lysis. The lysate was scraped and collected into 1.5-ml tubes and snap frozen on dry ice. Samples were subsequently processed as described below.

### Generation of internal standards

^15^N_1_^13^C_3_-labeled acyl-CoA internal standards were generated in yeast and extracted for use in quantitation experiments as described^60^. In brief, Pan6**Δ** *Saccharomyces cerevisiae* was cultured at 30 °C and 200 r.p.m. in synthetic defined media composed of yeast nitrogen base without amino acids and calcium pantothenate (Formedium, CYN3301) and drop-out mix complete w/o yeast nitrogen base (Formedium, DC50149) with the addition of ^15^N_1_^13^C_3_-calcium pantothenate 50 mM (CK Isotopes, CNLM-7694-0.01) for 72 h. Then, 1 mM sodium propionate (Sigma-Aldrich, P5436-100G) was added for the final 3 h to boost ^15^N_1_^13^C_3_-labeled propionyl-CoA generation. Cells were pelleted by centrifugation at 500 RCF at 4 °C for 10 min and extracted in ice-cold 10% (w/v) TCA. Cells were disrupted by sonication with a probe tip sonicator (60 × 0.5-s pulses at medium intensity). Protein was removed by centrifugation at 16,000*g* and 4 °C, and clarified supernatant containing internal standards was stored at −80 °C until use. Labeling efficiency was confirmed to be above 99% by MS analysis.

### Short chain acyl-CoA extraction

Cells and pre-weighed tissue samples were stored at −80 °C in 1.5-ml tubes before extraction. Samples were thawed on ice and kept on ice throughout processing. For quantitation experiments, an equal volume of internal standard was added to each sample across each experiment. The volume of internal standard used was adjusted to match the experimental sample type (for example, higher volume was used for tissue than for cell experiments to scale signal intensities of internal standards to unlabeled acyl-CoA in the sample). Standard curves were generated in parallel by the addition of an equal volume of internal standard to serial dilutions of acyl-CoA standard mixture. The acyl-CoA standard mixture stock contained succinyl-CoA and acetyl-CoA at 50 mM and other CoAs at 5 mM, as indicated in the table below. Internal standard was not added to isotope tracing samples. The samples were then sonicated for 10 × 0.5-s low-power pulses for cells but for 20 × 1-s high-power pulses for heart tissues. Protein was pelleted by centrifugation at 16,000*g* for 10 min and 4 °C. The supernatant was purified by solid-phase extraction using Oasis HLB 1-cc (30-mg) SPE columns (Waters). Columns were washed with 1 ml of methanol, equilibrated with 1 ml of water, loaded with supernatant of 10% TCA, desalted with 1 ml of water and eluted with 1 ml of methanol containing 25 mM ammonium acetate. The purified extracts were evaporated to dryness under nitrogen and then resuspended in 55 μl of 5% (w/v) 5-sulfosalicylic acid in water.

### Liquid chromatography

Analytes were separated by liquid chromatography on a Vanquish Duo UHPLC (Thermo Fisher Scientific) on a Waters XSelect Premier HSST3 2.5-μm particle size 2.1 × 100-mm column. A 5-μl sample aliquot was injected with the column temperature set at 25 °C and the flow rate set at 0.2 ml min^−1^. Two mobile phases were used for elution, including water with 5 mM ammonium acetate as phase A and 95:5 acetonitrile:water with 5 mM ammonium acetate as phase B. A third phase was used for column washing: 80:20 acetonitrile:water with 0.1% formic acid as phase C. The elution gradient (A,B) started with 0% phase B, was held for 2 min, was increased to 25% phase B at 5.5 min and then was increased to 100% phase B at 6 min and held until 14.5 min. At minute 14.5, flow was switched to 100% phase C and held until 19.5 min. Lastly, the gradient was switched to 2% phase B at 19.5 min and held until 25 min for re-equilibration. The sample manager temperature was set at 4 °C.

### Mass spectrometry

Mass spectrometry data acquisition was performed by using an Orbitrap IQ-X Tribrid mass spectrometer (Thermo Fisher Scientific). A heated electrospray ionization (ESI) source was used in positive ion mode. The spray voltage was set at 3.5 kV. The capillary temperature and aux gas heater temperature were set at 320 °C and 350 °C, respectively. Sheath gas and aux gas flow rate were set at 35 and 7 (in arbitrary units), respectively. The S-lens RF level was 60%. For MS1 full scan, the Orbitrap scan resolution was set at 120,000 (defined at 200 *m*/*z*), whereas data-dependent MS2 ion trap scan resolution was default at normal mode. The MS full scan range was 600–1,100 *m*/*z*, and the AGC target and maximum injection time were 400,000 and 50 ms, respectively. In addition, the MS2 scan range was 100–500 *m*/*z*, and the AGC target and maximum injection time were 10,000 and 35 ms, respectively. For data-dependent MS2 (ddMS2) mode, a targeted mass list was used as mass trigger for MS2 with a mass tolerance of 10 ppm for targeted molecules (indicated in Supplementary Table 10), and the isolation width of the precursor ion was set at 0.7 *m*/*z*. Other ddMS2 parameters were scan higher-energy collision dissociation (HCD) energy of 40%. Additionally, a dynamic exclusion filter was used to prevent ddMS2 of the same precursor ion whereby ddMS2 is excluded for 5 s if the same ions are detected three times in a row. Finally, an intensity filter of 1,000 (arbitrary units) was used for filtering background ions, alongside a precursor sort filter to prioritize the lower-intensity precursor ions.

### Data analysis

Full scan MS1 data were quantified by peak integration using TraceFinder version 5.1 (Thermo Fisher Scientific) software. ddMS2 fragmentation was used to validate characteristic neutral loss for CoA molecules using FreeStyle 1.8 SP2 (Thermo Fisher Scientific)^61^. Molecules were quantified as monoisotopic mass H^+^ (see Supplementary Table 10 for complete mass list). Retention times were matched to analytical standards in standard curves for further validation. For quantitation, relative area under the curve (AUC) was calculated by dividing AUC of analyte by the matching internal standard. If signal was insufficient for matching internal standard, a high-abundance internal standard at closest retention time was used instead. For absolute quantitation, standard curves were generated by plotting relative AUC against the known quantity of acyl-CoA analytical standards. Quantity in samples was interpolated within the linear range of the curve using GraphPad Prism software (version 9.4.0). Isotopic enrichment was calculated in tracing experiments by normalization to unlabeled control samples using the FluxFix calculator^62^. See Supplementary Table 11 for mass list for acyl-CoA analysis used in standard curves.

### Electrocardiography

Surface electrocardiograms were acquired from unconscious mice during the diurnal period using a non-invasive surgical monitoring platform (Indus Instruments, MouseMonitor^+^, courtesy of Carolyn Carr, University of Oxford). Anesthesia was induced in an anesthetic chamber by 2.5% isoflurane in oxygen and nitrous oxide (O_2_:N_2_O 4:1, total of 2 L min^−1^). Anesthesia was maintained at 1.8–2% for the duration of the experiment (O_2_:N_2_O 4:1, total of 2 L min^−1^). Conductive electrode cream (Indus Instruments, ECG Electrode Crème, 600-0001-01-S) was applied to the paws of the forelimb and hindlimb and secured onto the contact electrodes with hypoallergenic tape. Signals were grounded to the chassis and filtered as follows: high-pass filter 1 Hz, low-pass filter OFF and notch filter 50 Hz. Data were acquired using the manufacturer’s software in native format (.iird) and exported as .csv files. Downstream data analysis was performed in MATLAB. Electrocardiograms were recorded for 45 s, and an average over 30 s was analyzed to extract wave amplitudes (P, Q, R, S, J and T) relative to the isoelectric line and the duration between waves (RR, PR and QT). Recordings in animals with a core temperature below 35 °C during the recording or traces without a clear R wave were excluded.

### MR imaging

Cine MR imaging was performed in 8-week-old mice in the diurnal period as described previously^63,64^. Anesthesia was induced in an anesthetic chamber by 2.5% isoflurane in oxygen and nitrous oxide (O_2_:N_2_O 4:1, total of 2 L min^−1^). After induction, the mouse was transferred to the cradle positioned in the horizontal supine position, and anesthesia was maintained at 1.8–2% for the experiment (O_2_:N_2_O 4:1, total of 2 L min^−1^). The temperature of the cradle was maintained at 35 °C by warm airflow through an integrated heat exchanger to maintain core temperature at 37 °C. A bespoke electrocardiogram and a respiratory gating device were used to monitor heart and respiration signals. Electrocardiogram signals were obtained from electrodes inserted subcutaneously into the chest, and respiratory signals were obtained from a wire loop positioned loosely at the level of the sternum. A custom-made ^1^H/^13^C butterfly surface coil (loop diameter, 20 mm) was also placed over the mouse chest to localize the signal from the heart. The cradle was inserted into a horizontal-bore 7-T MR scanner interfaced to a direct-drive console with the animal’s heart positioned at the isocenter of the scanner. Correct positioning of the mouse was confirming by acquiring cardiac-triggered and respiration-gated axial FLASH (proton fast low-angle shot) images. Long-axis scans were taken to orientate the scanning angle and to set up true short-axis views. High-resolution cine MR short-axis images were then acquired in 9–10 contiguous slices (1 mm thick) covering the entire heart. Functional parameters of contractile function were derived from short-axis images. Each in vivo scanning protocol lasted 45–50 min. Animals were allowed to recover fully from anesthesia. For each 1-mm slice, end-diastolic and end-systolic frames were selected according to the size of the left ventricular (LV) lumen. For each frame, the epicardial and endocardial borders (drawing around any papillary muscles) were outlined using the free-hand tool in Fiji ImageJ. The epicardial and endocardial areas for each frame were summed across the *z*-stack to cover the entire heart to render LV end-diastolic and end-systolic volumes (EDVs and ESVs, respectively).

### Echocardiography

LV size and function were assessed in vivo by two-dimensional (2D) echocardiography in isoflurane-anesthetizsed mice using a high-resolution VisualSonics ultrasound system (Vevo 3100) with a 40-MHz transducer (MX400). Mice were maintained on 1–1.5% isoflurane in oxygen (1 L min^−1^) and placed on a homeothermic table, and parasternal short-axis B-mode views were obtained at the level of the papillary muscles. LV wall thickness and chamber dimensions were determined in the parasternal short-axis view (M-mode), from which LV structural and functional parameters were derived. 2D images of the heart were obtained from the apical four-chamber view to assess mitral blood flow (E and A) and tissue (e′ and a′) Doppler velocities. Body temperature, heart rate and respiratory rate were controlled during imaging, and all experiments were performed under physiological conditions. Data analysis was performed by a single operator blinded to genotype using VevoLAB (version 5.5.1). Measurements were made directly from the 2D images and are, therefore, expressed as areas.

### Fluorescence imaging of ventricular myocytes

To record CaTs, adult ventricular myocytes were AM-loaded for 10 min at room temperature with Fura Red (Invitrogen, F3021) at a concentration of 9.2 μM with 0.1% (w/v) Pluronic F-127. Ratiometric Ca^2+^ imaging was executed in dual-excitation mode (excitation alternating between 490 nm and 435 nm/emission 645 ± 37.5 nm). Fura Red ratios were calibrated to Ca^2+^ as described previously^39^. To measure cell size, adult ventricular myocytes were AM-loaded for 6 min at room temperature with cSNARF-1 (Invitrogen, C1272) at a concentration of 17.6 μM (excitation 555 nm/emission 580 nm and 640 nm). In neonatal ventricular myocytes, action potentials were imaged in cells loaded for 30 min at room temperature with FluoVolt (Invitrogen, F10488, 1:1,000 v/v, supplemented with Power-Load at 1:100 v/v) in single-excitation mode (excitation 488 nm/emission >510 nm). CaTs were imaged in neonatal ventricular myocytes AM-loaded for 10 min at room temperature with Fluo-3 (Invitrogen, F1242) at a concentration of 35.4 μM with 0.07% (w/v) Pluronic F-127 in single-excitation mode (excitation 488 nm/emission >510 nm).

Images were analysed to remove background and calculate the fluorescence ratio of the two wavelengths. An algorithm identified the CaT and caffeine responses and obtained the average CaT of a train for analysis. This provided measurements of diastolic, systolic, resting and peak-caffeine Ca^2+^ responses as well as timecourses of recovery required to calculate SERCA and NCX, as described previously^39^. Fluorescence images were also used to calculate cell shortening. This was obtained by thresholding the fluorescence map to identify the myocyte and measuring its area during the experimental protocol, followed by normalization to diastolic area.

Dual-excitation Fura Red imaging and single-excitation FluoVolt/Fluo-3 imaging was performed on an Olympus IX73 inverted microscope with an sCMOS camera (optiMOS, QImaging) and an LED light source (CoolLED, pE-4000), synchronized to the camera using a multichannel streaming device (MultiStream Pro, Cairn Research) and operated by MicroManager (version 1.4.23). cSNARF-1 imaging was performed in *x*–*y* mode on a Zeiss LSM 700 inverted confocal microscope. Adult ventricular myocytes were superfused at 37 °C with solution containing (in mmol L^−1^): 125 NaCl, 22 NaHCO_3_, 4.5 KCl, 1 MgCl_2_, 1 CaCl_2_, 11 glucose, 1 probenecid (5% CO_2_/air). Selected solutions contained 10 mM caffeine or 6 mM sodium propionate (osmolality balanced by adjusting (NaCl)). Neonatal ventricular myocytes were superfused at 37 °C with solution containing (in mmol L^−1^): 135 NaCl, 20 HEPES, 4.5 KCl, 1 MgCl_2_, 1 CaCl_2_, 11 glucose, 1 probenecid; pH adjusted to 7.40 at 37 °C with NaOH.

### Sulforhodamine B assay for cellular biomass

Cardiomyocyte hypertrophy was assessed for using a quantitative in vitro assay using sulforhodamine B (SRB)^65^. Isolated NRVMs were plated on specialized 96-well microplates (ibidi, μ-Plate 96-Well Black, 89626) at 60,000 cells per 200-μl well and treated for 48 h under serum-free conditions. After treatment, cells were rinsed in ice-cold PBS and fixed in 4% (w/v) paraformaldehyde/PBS (pH 7.4) for 10 min at 4 °C. The remaining steps were performed at room temperature. After fixation, cells were permeabilised in 0.5% (v/v) Triton X-100 for 10 min. Myocytes were then stained with the nuclear dye Hoechst 33342 (Invitrogen, H3570) for 5 min (0.1% v/v in PBS) before staining with 0.004% (w/v) SRB dissolved in 1% (v/v) acetic acid for 10 min. Wells were then rinsed four times with 1% acetic acid and dried. Plates were then imaged on a BioTek Cytation 5 plate reader system with a dry ×4 objective and sequential excitation at 377 nm and 531 nm. Fluorescence was then measured at 417–477 nm and 630–650 nm, respectively. The Hoechst and SRB images were analysed in MATLAB to quantify nuclear and extra-nuclear regions (nSRB and eSRB, respectively), and data were presented as the eSRB/nSRB ratio.

### Immunoblotting of histone extracts

Histones were extracted from cultured NRVMs using the EpiQuik Total Histone Extraction Kit (EpigenTek, OP-0006). Extracted histones were quantified using BCA assay (Pierce BCA Protein Assay Kit, Thermo Fisher Scientific, 23227) and stored in −80 °C until use. Samples were prepared in 4× Laemmli sample buffer (Bio-Rad) under reducing conditions (β-mercaptoethanol) and boiled at 100 °C for 5 min. SDS-PAGE was performed using pre-cast 4–20% gradient gels (Bio-Rad, Mini-PROTEAN TGX Precast Protein Gels, 4561096). Separated proteins were blotted onto PVDF membranes (Bio-Rad) for 90 min at 90 V. The PVDF membrane was blocked in 5% milk in 0.1% TBS-T (20 mM Tris-base, 137 mM NaCl, 0.1% v/v Tween 20, pH 7.40) for 1 h at room temperature. Primary antibodies were prepared in TBS-T with 1% BSA, and membranes were probed overnight at 4 °C. Primary antibodies used were against H3K9ac (Cell Signaling Technology, C5B11, 1:2,000), pan-pro-pionyllysine (PTM Biolabs, PTM201, 1:2,000), total H3 (Cell Signaling Technology, 1B1B2, 1:2,000) and total H4 (Cell Signaling Technology, D2X4V, 1:1,000). Blots were washed five times with TBS-T for 5 min and probed with HRP-conjugated secondary antibodies (Novus Biologicals) for 1 h at room temperature. After incubation with the secondaries, the TBS-T washes were repeated and blots were developed, using enhanced chemiluminescence (ECL) substrate and a ChemiDoc system (Bio-Rad).

### Immunofluorescence

Immunofluorescence was performed for histone modifications in the nuclei of ARVMs and NRVMs. Myocytes were plated on 12-well chamber glass slides (ibidi, 81201). Myocytes were fixed with ice-cold methanol for 10 min at 4 °C, and the remaining steps were performed at room temperature. After methanol fixation and before blocking, ARVMs were pre-treated with TrueBlack Lipofuscin Autofluorescence Quencher (Biotium, 23007) for 5 min at room temperature. Cells were blocked in PBS containing 3% BSA for 1 h. Primary antibodies were loaded in PBS/3% BSA for 90 min. Primary antibodies used were against H3K27ac (D5E4, Cell Signaling Technology, 1:100), total H3 (1B1B2, Cell Signaling Technology, 1:300) and vimentin (ab24525, Abcam, 1:300). Vimentin was used to identify any contaminant fibroblasts in NRVM preps. Cells were washed three times with PBS/0.05% Tween 20 and then incubated with the secondary antibodies in PBS/3% BSA for 1 h. After secondary antibody incubation, the wash step was repeated and the slide was covered, following application of anti-fade reagent (ProLong Gold Antifade, Invitrogen, P36934), with a cover glass (ibidi, 10811, 170-μm thickness). Secondary antibodies used were Alexa Fluor Plus 488-conjugated anti-rabbit IgG (A32731, Invitrogen, 1:1,000), Alexa Fluor Plus 555-conjugated anti-mouse IgG (A32727, Invitrogen, 1:1,000) and Alexa Fluor 405-conjugated anti-chicken IgY (ab175675, Abcam, 1:500). Selected coverslips were stained with Hoechst 33342 (0.1% v/v in PBS)—a DNA stain that emits blue fluorescence when excited at 405 nm (Invitrogen, H3570). Immunofluorescence was performed on a Zeiss LSM 700 confocal microscope with a ×40 oil immersion objective in *x–y* mode, with sequential excitation of wavelengths 405 nm, 488 nm and 555 nm.

### Whole-cell ELISA

Quantitative indirect ELISA was performed on fixed myocytes. NRVMs were seeded onto clear 96-well microplates at a density of 45,000 cells per well in culture media. At day 2 after isolation, myocytes were serum starved and, on day 3, treated for 24 h under serum-free conditions. After 24 h, cells were rinsed twice with cold 1× PBS and fixed with ice-cold methanol for 10 min at 4 °C, followed by a preliminary blocking stage with 0.3% H_2_O_2_ in PBS for 10 min at room temperature. Cells were blocked in blocking buffer containing 10% FCS in PBS-T (0.05% Tween 20) for 1 h at room temperature. Primary antibody was applied for 2 h at room temperature in blocking buffer. Primary antibodies were against H3K9ac (C5B11, Cell Signaling Technology, 1:300) and total H3 (1B1B2, Cell Signaling Technology, 1:300). Cells were then washed four times with PBS-T (0.05% Tween 20) and then incubated for 1 h at room temperature with HRP-conjugated secondary antibody in blocking buffer. Secondary antibodies were polymeric HRP goat α-rabbit IgG (ab214880, Abcam, 1:4) and polymeric HRP goat α-mouse IgG (ab214879, Abcam, 1:4). The PBS-T washing steps were repeated, and the ELISA was performed immediately. Development of the ELISA was based on McIlwain’s solution, containing 280 mM Na_2_HPO_4_ and 100 mM citric acid (pH 5.0 at room temperature). Immediately before use, H_2_O_2_ and o-phenylenediamine dihydrochloride (OPD) were added to McIlwain’s solution (10 mg of OPD and 10 μl of 30% H_2_O_2_ in 10 ml of McIlwain’s). Then, 50 μl of McIlwain’s development solution was added to each well, and the reaction was stopped by 100 μl of 2.0 M H_2_SO_4_. Absorbance was read immediately at 490 nm using a plate reader platform (Cytation 5, BioTek).

### RNA extraction

All procedures for RNA extraction were performed in DNase/RNase-free conditions. For NRVMs, the media was rinsed away with ice-cold PBS and RNA extracted by TRIzol reagent, according to the manufacturer’s protocol. Ventricular tissue from 8-week mice was snap frozen and cryoground. Bulk RNA was extracted using the Zymo Quick-RNA Mini-prep Plus Kit (R1057), following the manufacturer’s protocol with some modifications in the tissue digestion stage. All centrifugation steps were performed at 16,000 RCF and 4 °C. Then, 20 mg of cryoground tissue was mechanically homogenized on ice with disposable probes (Qiagen, TissueRuptor II) in 350 μl of DNA/RNA Shield. Next, 45 μl of proteinase K mix was added to 300 μl of sample, incubated for 30 min at 55 °C and centrifuged for 2 min after incubation. The supernatant was transferred to new microcentrifuge tubes, and 300 μl of RNA lysis buffer was added at 1:1 to the supernatant. Thereafter, the RNA purification procedure followed the manufacturer’s protocol with some differences: half-volume ethanol (300 μl) was added to isolate RNAs ≥200 nucleotides (nt) for poly(A) enrichment; DNase I treatment was performed in-column; and RNA was eluted in non-DEPC-treated nuclease-free water.

### RNA-seq

Library preparation and RNA-seq were performed by the Oxford Genomics Centre, Wellcome Centre for Human Genetics, University of Oxford. The assessment of RNA integrity was performed using the Agilent RNA 6000 Nano Kit with an Agilent 2100 Bioanalyzer. Samples were loaded onto a microplate (LVis Plate, BMG Labtech), and absorbance (*A*_260_/*A*_280_) was read using a SPECTROstar Nano plate reader (BMG Labtech). For library preparation, RNA was fractionated by enrichment for poly(A). Poly(A) + RNA was converted to a library of cDNA fragments and adapter ligated. Samples were sequenced on an Illumina NovaSeq 6000 system across 2 × 10-plex units to achieve an approximate sequencing depth of 25 million reads per sample. Raw reads were 150 bp in length. The following analysis was performed on a UNIX platform. Spliced Transcripts Alignment to a Reference (STAR) was used to align raw reads to the UCSC mouse reference genome *mm10* or *rn6* (ref. 66). PCR duplicates were removed. featureCounts was used to generate the list of DEGs and their abundance in the samples, expressed as counts per million (CPM)^67^. DEG analysis was performed using DESeq2 (ref. 68). The Wilkinson formula for analyzing mouse data was genotype+sex+genotype:sex. NRVM data were analyzed at three treatment levels (control, propionate and butyrate). After identifying the DEGs, the mouse and NRVM datasets were analysed and compared accordingly.

### RT–qPCR

RT–qPCR was performed in accordance with the MIQE guidelines^69^ and in DNase/RNase-free conditions. First-strand cDNA was synthesized using the High-Capacity cDNA Reverse Transcription Kit (Applied Biosystems, 4368814). RT–qPCR was performed on a ViiA 7 system (Life Technologies) using Fast SYBR Green (Fast SYBR Green 2× Master Mix, Applied Biosystems, 4385612). Each reaction used 4 ng of cDNA, and the concentration of primers was 200 nM. Fold changes were determined by the 2^−ΔΔCt^ method^70^. For mouse RT–qPCR experiments, genes of interest were normalized to the geometric mean of four reference genes (*Gusb, Pol2a, Tbp* and *Ywhaz*) previously described as stable in the heart^71^. For rat RT–qPCR experiments, genes of interest were normalized to *Hprt1*. Primers used are listed in Supplementary Table 12.

### ^13^C tracing histone MS/MS

Analyses were performed at the Target Discovery Institute at the University of Oxford.

### Sample preparation

Samples for ^13^C tracing metabolomics were obtained from NRVMs cultured with custom media as described above (‘isolation of neonatal ventricular myocytes’ and ‘^13^C tracing custom media’). Between 2 million and 2.5 million NRVMs were seeded per 60-mm Petri dish for each condition. For tracer experiments using U^13^C amino acids, NRVMs were treated for 48 h on day 3 after isolation, whereas NRVMs were treated for 24 h on day 4 after isolation for tracer experiments using 1-^13^C-propionate. For harvesting the NRVMs and isolation of intact histones, cells were trypsinised with 1× trypsin-EDTA and resuspended in ice-cold 1× PBS containing 10 mM sodium butyrate and protease inhibitor cocktails. The cell suspension was transferred to 1.5-ml tubes and centrifuged at 1,000 RCF for 2 min at 4 °C. The supernatant was discarded and the pellet resuspended in 150 μl of ice-cold hypotonic lysis buffer, containing (in mmol L^−1^): 10 Tris-HCl pH 8.0, 1 KCl, 1.5 MgCl_2_, 1 DTT, 10 sodium butyrate and protease inhibitor cocktail. The samples were incubated on a rotator for 30 min at 4 °C and then centrifuged at 10,000 RCF for 10 min at 4 °C. The supernatant was discarded. The pellet containing intact nuclei was resuspended in 100 μl of ice-cold 0.4 M HCl and incubated on a rotator for 1 h at 4 °C. Samples were then centrifuged at 16,000 RCF for 10 min at 4 °C, and the supernatant containing histones was transferred to 1.5-ml tubes. Histones were precipitated by adding 1 ml of ice-cold acetone to each sample, thoroughly mixing and then incubating overnight at −20 °C. The next day, histones were pelleted by centrifuging at 16,000 RCF for 10 min at 4 °C, and the pellet was washed with 1 ml of ice-cold acetone. Centrifugation was repeated at 16,000 RCF for 5 min at 4 °C; the supernatant was carefully removed; and the pellet was allowed to air-dry at room temperature for 20 min. Dried histone pellets were reconstituted in 25 μl of ultra-pure water and stored at −80 °C until further processing.

### Propionic anhydride derivatization and in-solution trypsin digestion

Histone protein concentration was quantified by BCA assay, and 20 μg of histone protein was used for sample preparation. D10-propionic anhydride derivatisation of histones was performed based on the H 42× hydroxylamine (HA) method from Meert et al.^72^ to cap unmodified lysines at the protein level. Propionic anhydride reagent for pre-trypsinisation propionylation was freshly prepared immediately before use by mixing D10-propionic anhydride (Eurisotop, DLM-3305-0.5) with isopropylalcohol at a 1:79 (v/v) ratio. Then, 20 μg of vacuum-dried histones (SpeedVac) was resuspended in 20 μl of 1 M TEAB (Merck, 18597), and an equal volume of propionic anhydride reagent was immediately added. After incubating at room temperature for 30 min, 20 μl of ultra-pure water was added and the sample incubated at 37 °C for 30 min. The sample was vacuum-dried and resuspended in 100 μl of buffer containing 50 mM TEAB, 1 mM CaCl_2_ and 5% ACN. Next, 100 μg of Trypsin Gold (Promega, V5280) was reconstituted in 200 μl of 50 mM ammonium bicarbonate, and trypsin was added to each sample at a histone:enzyme ratio of 20:1 and incubated overnight (16 h) at 37 °C. Samples were vacuum-dried and resuspended in 200 μl of 0.5 M hydroxylamine (Merck, 438227) to reverse overacylation. Then, 60 μl of ammonium hydroxide (Merck, 338818) was added to increase the pH to 12 (assessed with pH paper). The sample was then incubated at room temperature for 20 min and vacuum-dried until the total sample volume was less than 100 μl. Next, 25 μl of 20% TFA was added to lower the pH to ≤3 and proceed to sample clean-up. C18 desalting and clean-up was performed using Pierce C18 100-μl tips (Thermo Fisher Scientific, 87784). Tips were washed with 50% ACN and equilibrated with 0.1% TFA. Equilibrated tips were used to dispense and aspirate the sample ~10 times before rinsing tips with 0.1% TFA/5% ACN and eluting the peptides in 0.1% TFA/50% ACN into Protein LoBind tubes (Eppendorf). Samples were then dried down, and vacuum-dried peptides were stored in −20 °C until analysis.

### LC–MS/MS

The Orbitrap Fusion Lumos Tribrid mass spectrometer (Thermo Fisher Scientific) coupled to an Ultimate 3000 UHPLC (Thermo Fisher Scientific) was used to analyze the purified tryptic peptides. Six percent of tryptic peptides were loaded onto a trap column (PepMapC18, 300 μm × 5 mm, 5-μm particle size, Thermo Fisher Scientific) and separated on a 50-cm-long EASY-Spray column (ES803, Thermo Fisher Scientific) using a 60-min linear gradient from 2% to 35% buffer B (A: 5% DMSO, 0.1% formic acid; B: 5% DMSO, 0.1% formic acid in acetonitrile) at a 250 nl min^−1^ flow rate. Eluted peptides were then analysed on an Orbitrap Fusion Lumos Tribrid (instrument control software version 3.3). Data were acquired in data-dependent mode with the advance peak detection (APD) enabled. Survey scans were acquired in the Orbitrap at 120,000 resolution over an *m*/*z* range of 400–1,500, AGC target of 4 × 10^5^ ions and S-lens RF of 30. MS/MS spectra were obtained in the Orbitrap at 30,000 resolution with a quad isolation window of 1.6, AGC target of 5 × 10^4^ ions and maximum injection time of 54 ms, with HCD activation and a fixed collision energy set at 30%.

### Data analysis

Data were analysed using FragPipe version 19.1. Initially, the data were analyzed using the FragPipe open search work-flow to determine the amino acid mass shift induced by growing cells in the presence of heavy isoleucine and heavy valine or 1-C^13^-propionic acid as well as the mass shift induced by the in vitro derivatization at protein level of non-modified lysines with D10-propionic acid in the histone-enriched fractions (pre-trypsin digestion). The data were searched against the reviewed SwissProt–UniProt *Rattus norvegicus* proteome (downloaded on 3 February 2023, 10,102 sequences) and analysed using the default open search parameters. The open search identified mass shifts of +5.01677 in valine and +6.02129 in leucine as a result of the incorporation of heavy valine and isoleucine into proteins, respectively; +57.02956 in K corresponding to a K propionylation with 1-C^13^; +61.0574 in K and n-term corresponding to D10-propionylation and +59.03627 in K corresponding to propionylation with three C^13^ (3-Pro; probably coming from heavy valine or isoleucine). These mass shifts were used for a second FragPipe analysis. In this second FragPipe analysis, we used the Label Free Quantitation with Match Between Runs (LFQ-MBR) workflow with minor changes to the default settings. In brief, data were searched against the rat proteome, selecting trypsin as proteolytic enzyme (maximum of two missed cleavages) and oxidation (M; +15.9949), acetylation (K, N-terminus; +42.0106), heavy valine (+5.01677), heavy isoleucine (+6.02129), propionyl anhydride (K; 56.0262; Pro), propionyl:13C1 (K, +57.02956; 1-Pro), D10-propionic anhydride (k, N-terminus; +61.0574), propionyl mono-methyl (K, +70.0422; Pro-me) and heavy propionylation (k; +59.03627; 3-Pro) as variable modifications. MBR ion FDR of 1% was applied. Intensities and MaxLFQ intensities were reported in the data outputs. FragPipe modified-combined_peptides output was used to look at changes on histone propionylation induced by the experimental treatment. The mass spectrometry raw data related to histone PTMs were deposited to the ProteomeXchange Consortium via the PRIDE partner repository^73^ with the dataset identifier PXD043513.

### ChIP

For mouse ChIP, 8-week-old mouse hearts were fixed for 10 min at room temperature with 1% formaldehyde in modified adult mouse ventricular myocyte isolation solution containing 0.5 mM EGTA (solution pH 7.4 at room temperature) in Langendorff mode. Fixed ventricles were minced and rinsed twice in PBS and then lysed in ChIP lysis buffer (1% SDS, 10 mM Tris-HCl pH 8.0, 1 mM EDTA) by homogenisation (Qiagen, TissueRuptor II). For NRVM ChIP, NRVMs were fixed for 10 min with 1% formaldehyde in PBS and then lysed in ChIP lysis buffer. Samples were sonicated using a Covaris (Woburn) to achieve 200–300-bp fragments, according to the manufacturer’s protocol. For reference-normalized ChIP sequencing, fixed and sonicated *Drosophila* S2 cells were added to the sonicated samples at a ratio of 4:1 mouse/rat:*Drosophila*. Insoluble material was pelleted, and soluble sonicated chromatin was incubated overnight with antibodies against H3K27ac (Diagenode, C15410196), pan-propionyllysine (PTM Biolabs, PTM-201) or H3K23pr (Abcam, ab241466). Protein A/G Plus agarose beads (Thermo Fisher Scientific) were used to isolate antibody–chromatin complexes and then washed three times with RIPA buffer (50 mM HEPES-KOH pH 7.6, 500 mM LiCl, 1 mM EDTA, 1% NP-40, 0.7% Na deoxycholate) and once with TE-NaCl (10 mM Tris-HCl pH 8.0, 1 mM EDTA, 50 mM NaCl). Samples were treated with RNase A and proteinase K; crosslinks were reversed at 65 °C overnight; and then DNA was purified by phenol–chloroform extraction. For ChIP–qPCR, DNA was quantified with SYBR Green Master Mix (Thermo Fisher Scientific) relative to input chromatin. Primers used are listed in the table at the end of this section. Sequencing libraries for ChIP-seq were prepared using the NEBNext Ultra II DNA Library Preparation Kit (New England Biolabs), and 40-bp paired-end sequencing was conducted using a NextSeq 500 (Illumina). Bioinformatic analysis was conducted as previously described^74^. Sequencing quality was assessed with fastQC (http://www.bioinformatics.babraham.ac.uk/projects/fastqc/), and then reads were trimmed with trim_galore (https://www.bioinformatics.babraham.ac.uk/projects/trim_galore/) and mapped against rat, mouse or *Drosophila* genome assembly mm10, rn6 or dm6, respectively, using Bowtie2 (ref. 75). PCR duplicates were removed with Picard MarkDuplicates (http://broadinstitute.github.io/picard/). Sequence tag directories were generated using the Homer tool makeT-agDirectory, and then makeBigWig.pl was used to generate bigWig files for visualization in UCSC^76^. Peaks were called using findPeaks -style histone, with the input track providing background correction, and metagene profiles were generated using annotatePeaks.pl. Reference normalisation was achieved by calculating the ratio of dm6:mm10 or dm6:rn6 reads in input and immunoprecipitated samples, and used to scale bigWigs and metaplots. See Supplementary Table 13 for the list of primers.

### FRET

FRET imaging was performed as previously described^77^. NRVMs were transduced with second-generation adenoviral vectors encoding for the cGi500 FRET-based cGMP sensor^78^ on day 2 after isolation. FRET imaging was performed on an inverted microscope (Olympus IX71) using a PlanApoN, ×40, NA 1.42 oil immersion objective, 0.17/FN 26.5 (Olympus). The microscope was equipped with a CoolSNAP HQ2 monochrome camera (Photometrics) and an optical beam splitter device to simultaneously record YFP and CFP emissions (dual-view simultaneous imaging system, DV2, MAG Biosystems). FRET filter settings used throughout were: CFP excitation filter ET436/×20, dichroic mirror 455DCLP (Chroma Technology) in the microscope filter cube; dichroic mirror 505DCLP, YFP emission filter 545 nm and CFP emission filter 480 nm (Chroma Technology) in the beam splitter. Images were acquired and processed using Meta Imaging Series 7.1, MetaFluor (Molecular Devices). Fluorescence emission images were acquired every 5 s. Basal FRET was measured as the background-subtracted 480-nm/545-nm fluorescence emission intensity on excitation at 436 nm. FRET changes were measured as changes in the background-subtracted 480-nm/545-nm fluorescence emission intensity on excitation at 430 nm, where *R* is the 480-nm/545-nm value at time *t*. Cells were cultured on sterilised laminin-coated borosilicate glass coverslips, which allowed the transfer to a perfusion chamber. The solution for the measurements contained (in mmol L^−1^): 125 NaCl, 5 KCl, 1 Na_2_PO_4_, 1 MgSO_4_, 20 HEPES, 5.5 glucose and 1 CaCl_2_ (pH 7.4 at room temperature). Reagents used on experiments included: PF04449613 (PF9613, Bio-Techne Tocris), IBMX (Sigma-Aldrich/Merck), SNAP (Cayman Chemicals) and BAY41-2272 (Sigma-Aldrich/Merck).

### cGMP ELISA

cGMP concentration in mouse heart tissue was measured using a commercially available ELISA kit (Amersham cGMP Enzyme Immunoassay EIA Biotrak System, Cytiva, RPN226). The manufacturer´s protocol was used following ‘Protocol 2 – Acetylation EIA Procedure’ with some minor modifications. Then, 30 mg of snap-frozen ventricular tissue was homogenised on ice with 270 μl of cold 6% (w/v) TCA to give a 10% (w/v) homogenate. Samples were centrifuged at 2,000 RCF for 15 min at 4 °C. The pellet was kept for protein quantification (see below), and the supernatant was washed four times with 1 ml of water-saturated diethyl ether (prepared by mixing diethyl ether and ultra-pure water at a 1:1 ratio and vigorously mixing). The upper ether layer was discarded after each wash, and the remaining aqueous extract was vacuum-dried overnight (SpeedVac). The dried extract was resuspended in 1 ml of diluted assay buffer by mixing at 1,500 RPM (ThermoMixer, Eppendorf) for 5 min at room temperature. The resuspended mix was centrifuged briefly at 16,000 RCF for 30 s to clear insoluble debris, and the manufacturer´s protocol was followed for the remainder of the assay. A two-sample *F*-test for equal variance was used to test for statistical significance. To normalize measured cGMP concentrations to protein concentration, insoluble pellets were lysed in 130 μl of urea buffer (8 M urea, 1 M thiourea, 0.5% CHAPS, 50 mM DTT, 24 mM spermine) for 15 min at room temperature by mixing at 1,500 RPM (ThermoMixer). Total protein concentration was determined by Bradford assay.

### Phosphoproteomics

Analyses were performed in the Department of Biochemistry at the University of Oxford.

#### Sample preparation

Snap-frozen ventricular tissue was resuspended in 1 ml of lysis buffer (100 mM TEAB, 150 mM NaCl, 0.5% NP-40, protease and phosphatase inhibitors). The heart was thawed for 2–3 min before homogenising (Qiagen, TissueRuptor II). Homogenized sample was incubated on ice for 15 min and then centrifuged at 16,000 RCF for 30 min at 4 °C. Supernatant was collected containing whole-cell protein lysate, and protein concentration was determined using a spectrophotometer to measure absorbance at 280 nm (NanoDrop, Thermo Fisher Scientific). Then, 400 μg of protein was taken forward, and all samples were normalised to a total volume of 80 μl using lysis buffer. Detergent was removed using commercially available resin according to the manufacturer’s protocol (HiPPR Detergent Removal Resin, Thermo Fisher Scientific, 88305). Proteins were denatured by adding to the samples freshly prepared solution of 8 M urea in 100 mM ammonium bicarbonate (pH 7.8) at a 1:1 ratio. Samples were incubated for 10 min at room temperature with gentle shaking at 650 r.p.m. (ThermoMixer, Eppendorf). Cysteines were reduced with 10 mM TCEP (tris(2-carboxyethyl)phosphine) and incubated for 30 min at room temperature. ClAM (2-chloroacetamide) was freshly prepared in 8 M urea/100 mM ammonium bicarbonate solution and protected from light. Cysteines were then alkylated by adding 50 mM ClAM and incubating in the dark for 30 min at room temperature. Samples were pre-digested using LysC at a ratio of 1 μg of LysC to 100 μg of protein by incubating for 2 h at 37 °C with shaking at 800 r.p.m. The urea concentration was reduced to 2 M by diluting the sample with 100 mM ammonium bicarbonate solution, and CaCl_2_ was added to a final concentration of 2 mM. Next, 1 μg of trypsin was added for 100 μg of protein, and samples were digested overnight (16 h) at 37 °C with shaking at 800 r.p.m. Trypsin digestion was ceased by adding formic acid to a final concentration of 5%, and the samples were centrifuged at 16,000 RCF for 30 min at 4 °C to remove aggregates. The supernatant was collected and proceeded with desalting and sample clean-up using bespoke C18 columns produced using C18 resin and sterile 200-μl pipette tips. C18 resin was activated using 100% ACN, and the columns were equilibrated using 0.1% TFA. Peptides were eluted in two rounds of centrifugation using 100 μl of 50% ACN/0.1% TFA solution. Samples were transferred to Protein LoBind tubes and vacuum-dried overnight (SpeedVac). Phosphopeptide enrichment was performed using titanium dioxide (TiO_2_) microspin columns (TopTip, Glygen, TT2TIO) as described previously^79,80^.

#### LC–MS/MS

Peptides were separated by nano liquid chromatography (Thermo Fisher Scientific, Ultimate RSLC 3000 or EASY-nLC) coupled in line with a Q Exactive mass spectrometer equipped with an EASY-Spray source (Thermo Fisher Scientific). Peptides were trapped onto a C18 PepMac 100 pre-column (300 μm i.d. × 5 mm, 100 Å, Thermo Fisher Scientific) using solvent A (0.1% formic acid and HPLC-grade water). The peptides were further separated onto an EASY-Spray RSLC C18 column (75 μm i.d., 50-cm length, Thermo Fisher Scientific) using a 60-min linear gradient (15% to 35% solvent B (0.1% formic acid in acetonitrile)) at a flow rate of 200 nl min^−1^. The raw data were acquired on the mass spectrometer in data-dependent acquisition mode. Full-scan MS spectra were acquired in the Orbitrap (scan range 350–1,500 *m*/*z*, resolution 70,000; AGC target, 3 × 10^6^; maximum injection time, 50 ms). The 10 most intense peaks were selected for HCD fragmentation at 30% of normalized collision energy. HCD spectra were acquired in the Orbitrap at resolution 17,500, AGC target 5 × 10^4^ and maximum injection time 60 ms, with fixed mass at 180 *m*/*z*. Charge exclusion was selected for unassigned and 1+ ions. The dynamic exclusion was set to 40 s.

#### Data processing

MS/MS spectra were searched using Sequest HT in Proteome Discoverer software version 1.4 as follows. MS/MS data from TiO_2_-enriched samples were searched against a protein sequence database containing 17,962 protein entries, including 17,679 *Mus musculus* proteins (UniProt release from 28 March 2023) and 283 common contaminants. During database searching, cysteines (C) were considered to be fully carbamidomethylated (+57,0215, statically added), methionine (M) to be fully oxidized (+15,9949, dynamically added), all N-terminal residues to be acetylated (+42,0106, dynamically added) and serine (S), threonine (T) and threonine (Y) to be phosphorylated (+79,966, dynamically added). Two missed cleavages were permitted. Peptide mass tolerance was set at 20 ppm on the precursor and 0.6 Da on the fragment ions. Data were filtered at an FDR below 1% at peptide–spectrum match (PSM) level. Significant phosphorylated sequences were identified at 0.05 FDR and processed for kinase consensus site search (PhosphoSitePlus version 6.7). Intensity data for identified phospho-peptides was logged to base 2 before statistical analysis using a two-sample *t*-test to assess the difference in means between the two conditions. The data are displayed as a volcano plot with negative log_10_ of the *P* value on the *y* axis and difference between the means on the *x* axis. Thresholds of FDR < 0.05 with a log_2_(fold change) of >2 were used to determine significance of each phospho-site. Kinase substrates were annotated with putative kinases with log_2_(scores) better than 3. The mass spectrometry raw data related to phosphoproteomics were deposited to the ProteomeXchange Consortium via the PRIDE partner repository^73^ with the dataset identifier PXD043384.

### Statistics and data analysis

Statistical comparisons were made using paired or unpaired Student’s *t*-tests, repeated-measures or two-way ANOVA with Tukey’s post hoc tests, regression analyses or Pearson’s correlation analyses, as appropriate. Hierarchical statistical analysis was performed for cardiomyocyte data using R^81^ to avoid pseudo-replication error. *P* < 0.05 or *P*_adj_ < 0.05 was considered significant. Presented data represent mean ± s.e.m., unless stated otherwise.

### Reporting summary

Further information on research design is available in the Nature Portfolio Reporting Summary linked to this article.

## Extended Data

## Supplementary Material

Supplementary Tables 1-13

Supplementary Materials

## Data Availability

All data supporting the findings of this study are available in the supplementary materials or from the corresponding author upon reasonable request. High-throughput sequencing data were deposited in the Gene Expression Omnibus under accession code GSE205838. Mass spectrometry data were deposited to the ProteomeXchange Consortium via the PRIDE partner repository with the dataset identifier PXD043384 for phosphoproteomics data and the dataset identifier PXD043513 for histone proteomics data.
