# Supplementary material for: Disrupted propionate metabolism evokes transcriptional changes in the heart by increasing histone acetylation and propionylation
Source: Nat Cardiovasc Res. Author manuscript; Available in PMC 2024 Mar 18. (PMC7615744; doi:10.1038/s44161-023-00365-0)
Supplement: Supplementary Materials [file EMS194621-supplement-Supplementary_Materials.pdf]

nature portfolio

Corresponding author(s): Pawel Swietach

Last updated by author(s): Oct 6, 2023

Reporting Summary

Nature Portfolio wishes to improve the reproducibility of the work that we publish. This form provides structure for consistency and transparency in reporting. For further information on Nature Portfolio policies, see our Editorial Policies and the Editorial Policy Checklist.

Statistics

For all statistical analyses, confirm that the following items are present in the figure legend, table legend, main text, or Methods section.

n/a	Confirmed	
☐	☒	The exact sample size (*n*) for each experimental group/condition, given as a discrete number and unit of measurement
☐	☒	A statement on whether measurements were taken from distinct samples or whether the same sample was measured repeatedly
☐	☒	The statistical test(s) used AND whether they are one- or two-sided<br>*Only common tests should be described solely by name; describe more complex techniques in the Methods section.*
☐	☒	A description of all covariates tested
☐	☒	A description of any assumptions or corrections, such as tests of normality and adjustment for multiple comparisons
☐	☒	A full description of the statistical parameters including central tendency (e.g. means) or other basic estimates (e.g. regression coefficient) AND variation (e.g. standard deviation) or associated estimates of uncertainty (e.g. confidence intervals)
☐	☒	For null hypothesis testing, the test statistic (e.g. *F*, *t*, *r*) with confidence intervals, effect sizes, degrees of freedom and *P* value noted<br>*Give P values as exact values whenever suitable.*
☒	☐	For Bayesian analysis, information on the choice of priors and Markov chain Monte Carlo settings
☐	☒	For hierarchical and complex designs, identification of the appropriate level for tests and full reporting of outcomes
☐	☒	Estimates of effect sizes (e.g. Cohen's *d*, Pearson's *r*), indicating how they were calculated

Our web collection on statistics for biologists contains articles on many of the points above.

Software and code

Policy information about availability of computer code

Data collection

Raw metabolomics data files were collected and then processed using ProgenesisQI, Tracefinder or FluxFix calculator. Surface ECGs were acquired using Indus Instruments MouseMonitor+. Echocardiography scans were collected on a Vevo 3100 system. Cardiomyocyte Ca2+ transients were imaged and collected using MicroManager (v1.4.23). All plate reader assay data (e.g. SRB hypertrophy assay, ELISA) were collected from a Biotek Cytation 5 system. Immunoblots were developed and images collected on a ChemiDoc system (Bio-Rad). Immunofluorescence images were acquired using ZEN 2010B (Zeiss LSM 700). qPCR data were collected using QuantStudio real-time PCR software v1.3 (Applied Biosystems). RNA integrity was measured by an Agilent 2100 Bioanalyzer. Next-generation sequencing data (Illumina). Raw histone proteomics data were acquired with Orbitrap Fusion Lumos Tribrid instrument control software v3.3. Phosphoproteomics data were acquired using Sequest HT in Proteome discoverer software version 1.4.

Data analysis

Data analysis methods are described in the Supplement. The majority of data were analysed by a combination of MATLAB (R2021b), GraphPad Prism (v9.4.0) and R (v4.1.3).
For metabolomics, raw data files were processed using ProgenesisQI (Waters, Elstree, UK) and processed further with Metaboanalyst (https://www.metaboanalyst.ca/). Acyl-CoA data were analysed using FreeStyle 1.8 SP2 (Thermo Scientific), FluxFix calculator and Prism. Surface ECGs were using the monitoring platform native software (Indus Instruments MouseMonitor+). Cine-MRI images and Ca2+ transients were analysed in ImageJ (v1.5.2). Echocardiography data were analysed in VevoLAB (v5.5.1). FRET data were analysed by MetaFluor (Molecular Devices). Western blots were analysed using ImageLab (Biorad, v6.0.1).
Histone proteomics were analysed using FragPipe v19.1. Phosphoproteomics data were analysed using Phosphosite Plus v6.7.
For ChIP-seq and RNA-seq, sequencing quality was assessed with fastQC (http://www.bioinformatics.babraham.ac.uk/projects/fastqc/). RNA-seq raw reads were aligned with spliced Transcripts Alignment to a Reference (STAR) to the UCSC mouse reference genome mm10 or rn6. PCR duplicates were removed. featureCounts was used to generate the list of differentially expressed genes (DEGs) and their abundance in the samples, expressed as counts per million (cpm). DEG analysis was performed using DESeq2. ChIP-seq reads were trimmed with trim_galore

(https://www.bioinformatics.babraham.ac.uk/projects/trim_galore/) and mapped against rat, mouse or Drosophila genome assembly mm10, rn6 or dm6, respectively, using Bowtie2. PCR duplicates were removed with Picard MarkDuplicates (http://broadinstitute.github.io/picard/). Sequence tag directories were generated using the Homer tool makeTagDirectory, then makeBigWig.pl was used to generate bigwigs files for visualisation in UCSC.

For manuscripts utilizing custom algorithms or software that are central to the research but not yet described in published literature, software must be made available to editors and reviewers. We strongly encourage code deposition in a community repository (e.g. GitHub). See the Nature Portfolio guidelines for submitting code & software for further information.

Data

Policy information about availability of data

All manuscripts must include a data availability statement. This statement should provide the following information, where applicable:

- Accession codes, unique identifiers, or web links for publicly available datasets
- A description of any restrictions on data availability
- For clinical datasets or third party data, please ensure that the statement adheres to our policy

All data supporting the findings of this study are available in the Supplement or from the corresponding authors on reasonable request.

All newly-generated high throughput data have been deposited in the Gene Expression Omnibus (GEO) under the accession number GSE205838 (https://www.ncbi.nlm.nih.gov/geo/query/acc.cgi?acc=gse205838).
Listed below are the datasets associated with each figure:
Figure 5: Adult mouse RNA-seq (wild-type and PA, male and female in quintuplicate), NRVM RNA-seq (control, propionate and butyrate treatment in triplicate)
Figure 7: Adult mouse ChIP-seq for H3K27ac and pan-propionylation (wild type and PA female), NRVM ChIP-seq for H3K27ac (control and propionate treatment)

Mass spectrometry data have been deposited to the ProteomeXchange Consortium via the PRIDE partner repository with the dataset identifier PXD043384 for phosphoproteomics data and PXD043513 for histone proteomics data. Listed below are the datasets associated with each figure:
Figure 6: Phosphoproteomics in WT and PA female adult mouse hearts.
Figure 7: 13C tracing of propiogenic substrates and propionate to histone propionylation in NRVMs.

Human research participants

Policy information about studies involving human research participants and Sex and Gender in Research.

Reporting on sex and gender	*Use the terms sex (biological attribute) and gender (shaped by social and cultural circumstances) carefully in order to avoid confusing both terms. Indicate if findings apply to only one sex or gender; describe whether sex and gender were considered in study design whether sex and/or gender was determined based on self-reporting or assigned and methods used. Provide in the source data disaggregated sex and gender data where this information has been collected, and consent has been obtained for sharing of individual-level data; provide overall numbers in this Reporting Summary. Please state if this information has not been collected. Report sex- and gender-based analyses where performed, justify reasons for lack of sex- and gender-based analysis.*
Population characteristics	*Describe the covariate-relevant population characteristics of the human research participants (e.g. age, genotypic information, past and current diagnosis and treatment categories). If you filled out the behavioural & social sciences study design questions and have nothing to add here, write "See above."*
Recruitment	*Describe how participants were recruited. Outline any potential self-selection bias or other biases that may be present and how these are likely to impact results.*
Ethics oversight	*Identify the organization(s) that approved the study protocol.*

Note that full information on the approval of the study protocol must also be provided in the manuscript.

Field-specific reporting

Please select the one below that is the best fit for your research. If you are not sure, read the appropriate sections before making your selection.

☒ Life sciences ☐ Behavioural & social sciences ☐ Ecological, evolutionary & environmental sciences

For a reference copy of the document with all sections, see nature.com/documents/nr-reporting-summary-flat.pdf

Life sciences study design

All studies must disclose on these points even when the disclosure is negative.

Sample size	The sample sizes were based on previously published studies (Nyamundanda et al. 2013, PMID 24261687; Sikkel et al. 2017, PMID 29016722;

Sample size	Guo et al. 2014, PMID 25374457) as well as other studies in the field, which provide sufficient sample numbers to statistically detect differences between multiple experimental groups.
Data exclusions	For Ca2+ transient experiments, any cardiomyocytes that had spontaneous Ca2+ release events during the recording protocol were excluded. No other data were excluded in the study.
Replication	Experiments in this study were conducted on different days using different animals. The experimental data were analysed statistically. For cardiomyocyte studies, hierarchical statistical analysis was performed using previously published methods to avoid pseudoreplication error and to test reproducibility. Assay-based studies were run with biological replicates, each with technical replicates.
Randomization	Animal/sample processing was performed in a randomised order where possible.
Blinding	Blinding was performed where possible during this study (e.g. during analysis), however the majority of this study was not blinded due to experiments being conducted by the same researcher.

Reporting for specific materials, systems and methods

We require information from authors about some types of materials, experimental systems and methods used in many studies. Here, indicate whether each material, system or method listed is relevant to your study. If you are not sure if a list item applies to your research, read the appropriate section before selecting a response.

Materials & experimental systems

n/a	Involved in the study	
☐	☒	Antibodies
☒	☐	Eukaryotic cell lines
☒	☐	Palaeontology and archaeology
☐	☒	Animals and other organisms
☒	☐	Clinical data
☒	☐	Dual use research of concern

Methods

n/a	Involved in the study	
☐	☒	ChIP-seq
☒	☐	Flow cytometry
☒	☐	MRI-based neuroimaging

Antibodies

Antibodies used	Anti-histone H3K27ac (ChIP-seq and ChIP-qPCR): Diagenode C15410196. ChIP-seq [1:350], ChIP-qPCR [1:1000].<br>Anti-pan-propionylation (ChIP-seq, ChIP-qPCR, WB): PTM Biolabs PTM201. ChIP-seq [1:500], ChIP-qPCR [1:2000], WB [1:2000].<br>Anti-Histone H3K23pr (ChIP-qPCR): Abcam ab241466. ChIP-qPCR [1:500].<br>Anti-histone H3K9ac (WB, IF, ELISA): Cell Signalling Technology 9649, clone C5B11. ELISA and IF [1:300], WB [1:2000].<br>Anti-histone H3K27ac (IF): Cell Signalling Technology 8173, clone D5E4. IF [1:100].<br>Anti-histone H3 (WB, IF, ELISA): Cell Signalling Technology 14269, clone 1B1B2. ELISA and IF [1:300], WB [1:2000].
Validation	Anti-histone H3K27ac (ChIP-seq and ChIP-qPCR): Diagenode C15410196. Validated for ChIP on the manufacturer's website and in-house.<br>Anti-pan-propionylation (ChIP-seq, ChIP-qPCR, WB): PTM Biolabs PTM201. Validated by dot-blot and WB on the manufacturer's website.<br>Anti-Histone H3K23pr (ChIP-qPCR): Abcam ab241466. Validated by IHC, ELISA and WB on the manufacturer's website.<br>Anti-histone H3K9ac (WB, IF, ELISA): Cell Signalling Technology 9649, clone C5B11. Validated for IF on the manufacturer's website.<br>Anti-histone H3K27ac (IF): Cell Signalling Technology 8173, clone D5E4. Validated for IF on the manufacturer's website.<br>Anti-histone H3 (WB, IF, ELISA): Cell Signalling Technology 14269, clone 1B1B2. Validated for IF and WB on the manufacturer's website.

Animals and other research organisms

Policy information about studies involving animals; ARRIVE guidelines recommended for reporting animal research, and Sex and Gender in Research

Laboratory animals	In vivo model of elevated propionate: all mouse experiments were performed on adult mice that were 8 weeks of age from the Pcca-/- A138T line on an FVB background, generated by Guenzel et al 2013 Mol Ther. Mice used were either wild-types (Pcca+/+ A138T) for control experiments, or homozygous (Pcca-/- A138T) animals, referred to as amWT and amPA respectively in this study.<br><br>In vitro cardiomyocyte models: experiments were performed on ventricular myocytes either isolated from 275-300g adult or post-natal day 1-2 (P1/P2) neonatal Sprague-Dawley rats (Charles River Laboratories).
Wild animals	This study did not involve wild animals.
Reporting on sex	Sex was considered in the design of this study and the majority of the experiments were performed sex-balanced; single-sex studies were performed in particular experiments and these are indicated appropriately. Sex was considered in statistical analyses in sex-

balanced experiments.

Field-collected samples	Field-collected samples were not involved in this study.
Ethics oversight	All protocols were conducted in accordance with local guidelines (University of Oxford) and the UK Animals (Scientific Procedures) Act 1986.

Note that full information on the approval of the study protocol must also be provided in the manuscript.

ChIP-seq

Data deposition

☒ Confirm that both raw and final processed data have been deposited in a public database such as GEO.

☒ Confirm that you have deposited or provided access to graph files (e.g. BED files) for the called peaks.

Data access links
May remain private before publication.

To review GEO accession GSE205838:
Go to https://www.ncbi.nlm.nih.gov/geo/query/acc.cgi?acc= GSE205838

Files in database submission

amWT-PA_RNA_mm10_featuresCounted.txt
amWT-PA_RNA_mm10_female_contrast_cpm.csv
amWT-PA_RNA_mm10_male_contrast_cpm.csv
RNA_amWT-F_rep1_R1.fastq.gz, RNA_amWT-F_rep1_R2.fastq.gz
RNA_amWT-F_rep2_R1.fastq.gz, RNA_amWT-F_rep2_R2.fastq.gz
RNA_amWT-F_rep3_R1.fastq.gz, RNA_amWT-F_rep3_R2.fastq.gz
RNA_amWT-F_rep4_R1.fastq.gz, RNA_amWT-F_rep4_R2.fastq.gz
RNA_amWT-F_rep5_R1.fastq.gz, RNA_amWT-F_rep5_R2.fastq.gz
RNA_amPA-F_rep1_R1.fastq.gz, RNA_amPA-F_rep1_R2.fastq.gz
RNA_amPA-F_rep2_R1.fastq.gz, RNA_amPA-F_rep2_R2.fastq.gz
RNA_amPA-F_rep3_R1.fastq.gz, RNA_amPA-F_rep3_R2.fastq.gz
RNA_amPA-F_rep4_R1.fastq.gz, RNA_amPA-F_rep4_R2.fastq.gz
RNA_amPA-F_rep5_R1.fastq.gz, RNA_amPA-F_rep5_R2.fastq.gz
RNA_amWT-M_rep1_R1.fastq.gz, RNA_amWT-M_rep1_R2.fastq.gz
RNA_amWT-M_rep2_R1.fastq.gz, RNA_amWT-M_rep2_R2.fastq.gz
RNA_amWT-M_rep3_R1.fastq.gz, RNA_amWT-M_rep3_R2.fastq.gz
RNA_amWT-M_rep4_R1.fastq.gz, RNA_amWT-M_rep4_R2.fastq.gz
RNA_amWT-M_rep5_R1.fastq.gz, RNA_amWT-M_rep5_R2.fastq.gz
RNA_amPA-M_rep1_R1.fastq.gz, RNA_amPA-M_rep1_R2.fastq.gz
RNA_amPA-M_rep2_R1.fastq.gz, RNA_amPA-M_rep2_R2.fastq.gz
RNA_amPA-M_rep3_R1.fastq.gz, RNA_amPA-M_rep3_R2.fastq.gz
RNA_amPA-M_rep4_R1.fastq.gz, RNA_amPA-M_rep4_R2.fastq.gz
RNA_amPA-M_rep5_R1.fastq.gz, RNA_amPA-M_rep5_R2.fastq.gz
NRVM_RNA_rn6_featuresCounted.txt
NRVM_RNA_rn6_PRO_contrast_cpm.csv
NRVM_RNA_rn6_BUT_contrast_cpm.csv
RNA_NRVM-CON_rep1_R1.fastq.gz, RNA_NRVM-CON_rep1_R2.fastq.gz
RNA_NRVM-CON_rep2_R1.fastq.gz, RNA_NRVM-CON_rep2_R2.fastq.gz
RNA_NRVM-CON_rep3_R1.fastq.gz, RNA_NRVM-CON_rep3_R2.fastq.gz
RNA_NRVM-PRO_rep1_R1.fastq.gz, RNA_NRVM-PRO_rep1_R2.fastq.gz
RNA_NRVM-PRO_rep2_R1.fastq.gz, RNA_NRVM-PRO_rep2_R2.fastq.gz
RNA_NRVM-PRO_rep3_R1.fastq.gz, RNA_NRVM-PRO_rep3_R2.fastq.gz
RNA_NRVM-BUT_rep1_R1.fastq.gz, RNA_NRVM-BUT_rep1_R2.fastq.gz
RNA_NRVM-BUT_rep2_R1.fastq.gz, RNA_NRVM-BUT_rep2_R2.fastq.gz
RNA_NRVM-BUT_rep3_R1.fastq.gz, RNA_NRVM-BUT_rep3_R2.fastq.gz
amWT-F_H3K27ac.bigWig, amWT-F_H3K27ac-input.bigWig
amWT-F_Kpr.bigWig, amWT-F_Kpr-input.bigWig
amPA-F_H3K27ac.bigWig, amPA-F_H3K27ac-input.bigWig
amPA-F_Kpr.bigWig, amPA-F_Kpr-input.bigWig
amWT-F_H3K27ac_R1.fastq.gz, amWT-F_H3K27ac_R2.fastq.gz
amWT-F_H3K27ac-input_R1.fastq.gz, amWT-F_H3K27ac-input_R2.fastq.gz
amWT-F_Kpr_R1.fastq.gz, amWT-F_Kpr_R2.fastq.gz
amWT-F_Kpr-input_R1.fastq.gz, amWT-F_Kpr-input_R2.fastq.gz
amPA-F_H3K27ac_R1.fastq.gz, amPA-F_H3K27ac_R2.fastq.gz
amPA-F_H3K27ac-input_R1.fastq.gz, amPA-F_H3K27ac-input_R2.fastq.gz
amPA-F_Kpr_R1.fastq.gz, amPA-F_Kpr_R2.fastq.gz
amPA-F_Kpr-input_R1.fastq.gz, amPA-F_Kpr-input_R2.fastq.gz
NRVM-CON_H3K27ac.bigWig, NRVM-CON_H3K27ac-input.bigWig
NRVM-PRO_H3K27ac.bigWig, NRVM-PRO_H3K27ac-input.bigWig
NRVM-CON_H3K27ac_R1.fastq.gz, NRVM-CON_H3K27ac_R2.fastq.gz
NRVM-CON_H3K27ac-input_R1.fastq.gz, NRVM-CON_H3K27ac-input_R2.fastq.gz
NRVM-PRO_H3K27ac_R1.fastq.gz, NRVM-PRO_H3K27ac_R2.fastq.gz
NRVM-PRO_H3K27ac-input_R1.fastq.gz, NRVM-PRO_H3K27ac-input_R2.fastq.gz

Genome browser session
(e.g. UCSC)

https://genome.ucsc.edu/s/ncrump/amPA_review
https://genome.ucsc.edu/s/ncrump/NRVM_review

Methodology

Replicates

One replicate for ChIP-seq datasets.

Sequencing depth

amWT H3K27ac: total reads: 44112157, uniquely mapped reads: 34071697, read length: 40 bp, Paired end
amPA H3K27ac: total reads: 37345576, uniquely mapped reads: 31042196, read length: 40 bp, Paired end
amWT H3K27ac input: total reads: 29301783, uniquely mapped reads: 27280374, read length: 40 bp, Paired end
amPA H3K27ac input: total reads: 30163897, uniquely mapped reads: 27884191, read length: 40 bp, Paired end
amWT Kpr: total reads: 37270422, uniquely mapped reads: 33489664, read length: 40 bp, Paired end
amPA Kpr: total reads: 28635044, uniquely mapped reads: 25901959, read length: 40 bp, Paired end
amWT Kpr input: total reads: 29462563, uniquely mapped reads: 27723503, read length: 40 bp, Paired end
amPA Kpr input: total reads: 9292088, uniquely mapped reads: 7730453, read length: 40 bp, Paired end
NRVM control H3K27ac: total reads: 38913036, uniquely mapped reads: 32250722, read length: 40 bp, Paired end
NRVM propionate H3K27ac: total reads: 37957220, uniquely mapped reads: 33777326, read length: 40 bp, Paired end
NRVM control H3K27ac input: total reads: 32574208, uniquely mapped reads: 29155469, read length: 40 bp, Paired end
NRVM propionate H3K27ac input: total reads: 30930488, uniquely mapped reads: 27263596, read length: 40 bp, Paired end

Antibodies

Anti-histone H3K27ac: Diagenode C15410196, lot A1723-0041D.
Anti-pan-propionyllysine: PTM Biolabs PTM201, lot Z033L0323P3.

Peak calling parameters

Peaks were called using the Homer tool findPeaks, with the input track provided for background correction, using the -style histone option.

Data quality

Reads were filtered to remove PCR duplicates. Called peaks were analyzed as described on homer.ucsd.edu and compared to input track, with a threshold of FDR < 0.001 applied.

Software

Sequencing quality was assessed with fastQC (http://www.bioinformatics.babraham.ac.uk/projects/fastqc/), then reads were trimmed with trim_galore (https://www.bioinformatics.babraham.ac.uk/projects/trim_galore/) and mapped against rat, mouse or Drosophila genome assembly mm10, rn6 or dm6, respectively, using Bowtie2. PCR duplicates were removed with Picard MarkDuplicates (http://broadinstitute.github.io/picard/). Sequence tag directories were generated using the Homer tool makeTagDirectory, then makeBigWig.pl was used to generate bigwig files for visualisation in UCSC.
